# Micro-arc oxidation (MAO) and its potential for improving the performance of titanium implants in biomedical applications

**DOI:** 10.3389/fbioe.2023.1282590

**Published:** 2023-11-07

**Authors:** Xueying Wen, Yan Liu, Fangquan Xi, Xingwan Zhang, Yuanyuan Kang

**Affiliations:** ^1^ School and Hospital of Stomatology, China Medical University, Liaoning Provincial Key Laboratory of Oral Diseases, Shenyang, China; ^2^ School of Mechanical Engineering and Automation, Northeastern University, Shenyang, China

**Keywords:** Ti and its alloys, micro-arc oxidation, biological properties, antibacterial properties, coating

## Abstract

Titanium (Ti) and its alloys have good biocompatibility, mechanical properties and corrosion resistance, making them attractive for biomedical applications. However, their biological inertness and lack of antimicrobial properties may compromise the success of implants. In this review, the potential of micro-arc oxidation (MAO) technology to create bioactive coatings on Ti implants is discussed. The review covers the following aspects: 1) different factors, such as electrolyte, voltage and current, affect the properties of MAO coatings; 2) MAO coatings affect biocompatibility, including cytocompatibility, hemocompatibility, angiogenic activity, corrosion resistance, osteogenic activity and osseointegration; 3) antibacterial properties can be achieved by adding copper (Cu), silver (Ag), zinc (Zn) and other elements to achieve antimicrobial properties; and 4) MAO can be combined with other physical and chemical techniques to enhance the performance of MAO coatings. It is concluded that MAO coatings offer new opportunities for improving the use of Ti and its alloys in biomedical applications, and some suggestions for future research are provided.

## 1 Introduction

Metal implants have been a staple in biomedical applications since the onset of the 19th century, with Ti and its alloys becoming the prime choice for medical implant materials with high biocompatibility, excellent corrosion resistance, high specific strength and the best osteogenic potential (Kaur and Singh, 2019). Since the mid-twentieth century, due to their mechanical and chemical properties, titanium and its alloys have been widely used in industrial and biomedical applications, particularly in dentistry and orthopaedics such as dental implants, bone fusion, bone fixation and arthroplasty (joint replacement surgery) (Anderson et al., 2021; Hoque et al., 2022; Lim et al., 2022).

Although titanium and its alloys have been extensively used, they are inert metals that do not effectively promote the proliferation of osteoblasts and osteocytes, which can lead to implant failure. In addition, titanium-based implants still face some clinical problems, such as slow biological reactions to the material surface, slow early bone integration, and implantable diseases caused by bacterial colonization, leading to implant loosening and survival ability loss (Zhang et al., 2020b). Surface morphology, composition, hydrophilicity and roughness are key factors of implant tissue interaction and osseointegration (Le Guéhennec et al., 2007). The bioactive calcium phosphate (Ca-P)-containing coatings on titanium are similar to biomimetic implant materials used for bone tissue. Therefore, we can focus on the development of Ca-P-based surface coatings on titanium-based materials for load-bearing implant applications. Hence, typical coating methodologies have emerged in recent years. The methodologies can be divided into three categories: mechanical methods (fine machining, grinding, tumbling, and sandblasting), physical methods [physical vapour deposition (PVD), thermal spraying, and ion implantation] and chemical methods [pickling, chemical vapour deposition (CVD), anodic oxidation, sol-gel, and micro-arc oxidation (MAO)] (Shen et al., 2019; Gabor et al., 2020). These methods can improve the properties of titanium and its alloys, such as their better wear and corrosion resistance, providing strong mechanical adhesion between bone and implant, enhancing osteo-induction and osteo-conduction, improving bioactivity and biocompatibility, and accelerating healing time at the implant site (Sasikumar et al., 2019; Xue et al., 2020). At the same time, they have different advantages and disadvantages (as shown in Table 1).

**TABLE 1:** Surface treatment methods with their advantages, disadvantages, and applications

Method	Advantages	Disadvantages	Ref.
Sandblasting	Enhancing implant surface roughness and biocompatibility	Sandblasting particles and etchants may not be completely removed, leading to surface residues	Szmukler-Moncler et al. (2004); Wennerberg and Albrektsson (2009); Shemtov-Yona et al. (2014); Velasco et al. (2016)
	Increased cell attachment, promoted osseointegration and bone bonding	Enhancing bacterial adhesion and accumulation	
	Improving initial stability of the implant	Uncontrolled sandblasting may result in surface cracking of the implant	
	Increased the fatigue test lifespan of the implant	Improper sandblasting speed or particle size may reduce the implant's lifespan	
PVD	Improved the hardness and wear resistance of the coating	Not suitable for materials with high melting points and relatively low melting points, such as Al and Mg	Kaseem et al. (2021)
	Improved biological activity	Coatings usually have low thickness and poor adhesion	
	Improve bonding strength	The process is complex, takes a long time uneconomical	
Thermal spraying	Dense and high-temperature oxidation-resistant coating	High cost	Liu et al. (2020); Xue et al. (2020)
	High bonding strength convenient	Difficult to coat the inner surface of small holes	
	Enhanced wear-resistant, corrosion-resistant, and oxidation-resistant		
	Coating can accelerate bone regeneration and has good biocompatibility		
Ion implantation	Improve the tribological properties and corrosion resistance of metals	May release metal ions, causing cytotoxicity	Rautray et al. (2010); Wang et al. (2014); Xue et al. (2020)
	Selectively modify the surface without adversely affecting the overall performance		
	Extremely high controllability and repeatability		
	High accuracy in controlling the concentration and depth distribution of impurities		
Chemical vapour deposition	Improved corrosion resistance	Poor coverage over large areas and complex surfaces	Antunes and de Oliveira (2009)
	Wear resistance and fatigue resistance	The thickness of the coating may be uneven	
	High purity, easy to control and monitor	High radiant heat may change the properties of the substrate material	
	High vaporization rate		
	Relatively low cost		
Anodic oxidation	Enhance coating hardness and thickness	Not environmentally friendly	Hu et al. (2021); Seyidaliyeva et al. (2022); Wang et al. (2022)
	Alter coating colour	Slow deposition rate	
	Improve the adhesion and abrasion resistance of coating	Strict pre-treatment requirements	
	Inexpensive		
Sol-gel method	Simple manufacturing environment and reliable equipment	High Cost	Hsieh et al. (2002); Gan and Pilliar (2004); Ishikawa et al. (2020); Azzouz et al. (2022)
	High uniformity of the coatings	Long process time	
	Applicability to substrates of different sizes	Poor adhesion to a substrate	
	Significant enhancement of corrosion protection and biocompatibility of the metal substrate, reducing the risk of implant rejection	Require postprocessing to obtain more desirable coating properties	
	Ease of controlling chemical composition and microstructure		
	Suitable for coating complex-shaped substrates		
	High preparation efficiency		
Micro-arc oxidation	The coating has excellent corrosion resistance, high hardness, good adhesion, and is evenly distributed	Exhibit minor defects such as micro-cracks, micro-pores, and discharge channels	Pesode and Barve (2021); Li et al. (2023)
	Suitable for complex surfaces,	Rapid increases in the electrolyte temperature can make it challenging to maintain stable control over the micro-arc oxidation process	
	Easy to process and control	Excessively high electrolyte temperatures may result in over-discharge, leading to uneven surface topography of the coating	
	Low-cost and cost-effective	Relatively high energy consumption	

MAO [plasma electrolytic oxidation (PEO), or anodic spark deposition (ASD)] has attracted substantial attention as an emerging surface coating methodology. MAO uses an arc discharge to enhance and activate the reactions occurring on an anode to form a ceramic film on the surface of a metal through the interaction of the workpiece and electrolyte (Wang Y. et al., 2015; Kaseem and Ko, 2019b; Kaseem and Ko, 2019a; Zhang R. et al., 2021; Xia et al., 2021). This approach can incorporate bioactive electrolyte components such as Ca and P into the coating. Ca-P coatings, particularly HA coatings, can facilitate the process of early and rapid osseointegration. The bioactivity of the coating is determined by its physicochemical characteristics, including roughness, porosity, phase, elemental composition, and adhesive strength (Wang et al., 2022).

In addition, the process of MAO can be regulated by multiple factors. On the one hand, a porous bioactive Ca-P-based composite layer can be deposited on Ti-based implant surfaces according to the selected electrolyte, which would enhance the biocompatibility and bonding strength of the coated layer (Zhao et al., 2007; Sun et al., 2008). On the other hand, antibacterial metal elements can be incorporated into implant surfaces to inhibit the initial adhesion of bacteria and prevent postsurgical complications, thus enhancing the antibacterial properties (Ferraris and Spriano, 2016). Furthermore, the contents of bioactive elements and antibacterial metal elements on the MAO coating surface can be tuned by controlling the voltage, electrolyte components and MAO time.

This review aims to collect and compare recent scientific papers concerning Ti and its alloys modified by MAO technology in the biomedical field. This review is focused on several aspects, including the influences of different factors on the performance of MAO coatings, the biocompatibility of MAO coatings and the combination of MAO with other physical or chemical techniques. In addition, implant-associated infection remains one of the most devastating postoperative complications (Oh-Hyun et al., 2018). It is highly desirable to introduce antimicrobial agents into implant surfaces to provide antibacterial activities and prevent peri-implant infections. Due to their perfect stabilities, superior broad-spectrum antibacterial properties and relatively low toxicity levels, inorganic antibacterial metal elements [e.g., silver (Ag), copper (Cu) and zinc (Zn)] have attracted great attention, which was elaborated in detail in our review. Finally, we highlight the potential challenges and future applications of MAO-modified titanium and its alloys in the biomedical field.

## 2 MAO process parameters for Ti and its alloys

**FIGURE 1:**
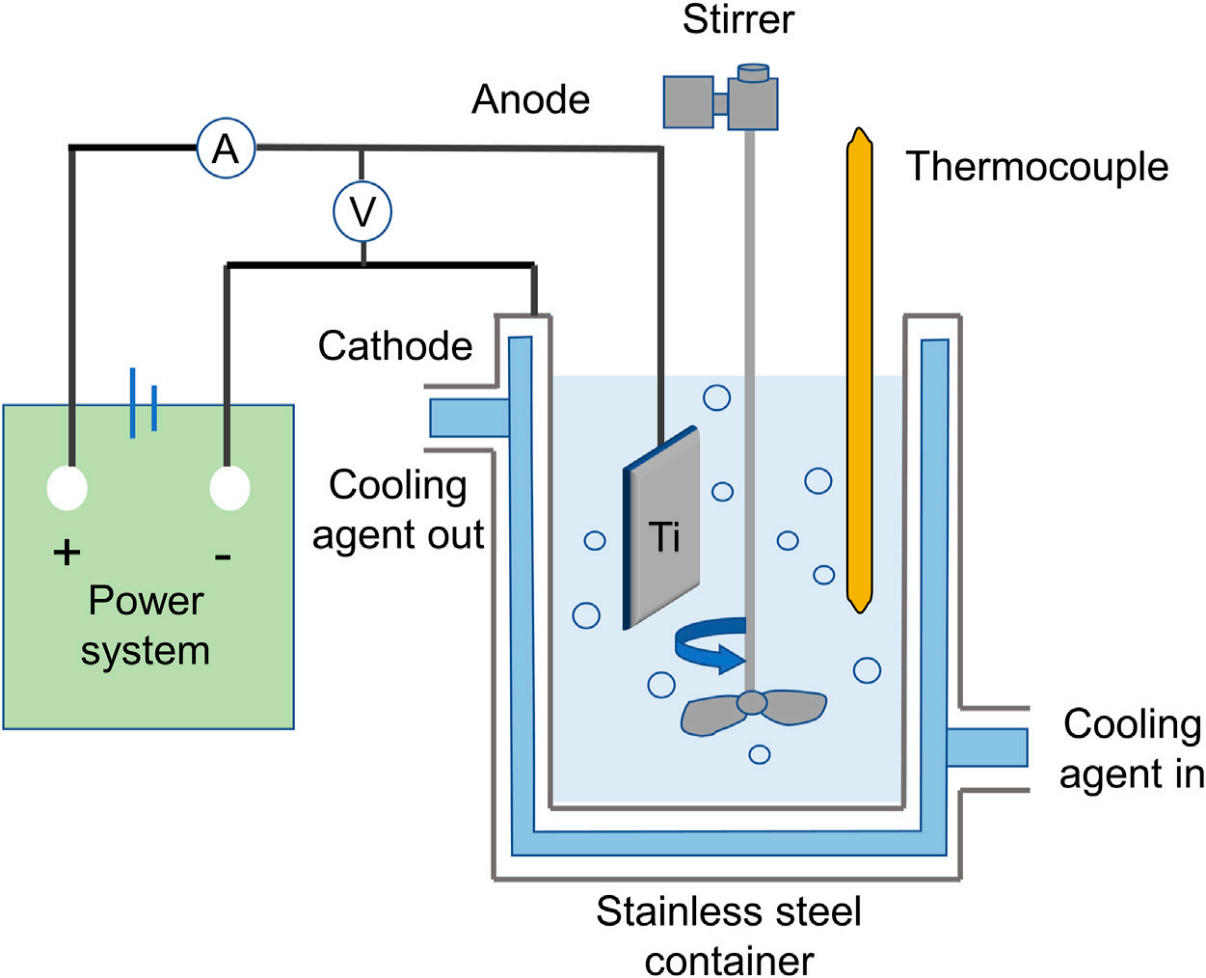
Schematic representation of the MAO coating system.

Although more research is needed to fully understand the mechanisms at play, experts have identified three key components of the MAO process: electrochemical oxidation, plasma chemistry, and thermal diffusion in the electrolyte (Hussein et al., 2013). During this process, Ti and its alloys are submerged in an electrolyte containing modified species in the form of dissolved salts, such as silicates (Si), phosphates(P) and Ca salts (Sarbishei et al., 2014). The Ti and its alloys are used as the anode, while stainless steel plates serve as the cathode in the electrolytic bath (as depicted in Figure 1) Typically, MAO treatments last between 5 and 180 min, with current densities ranging from 500 to 2,000 A-m-2 and voltages reaching up to 1,000 V (Snizhko et al., 2004).

**FIGURE 2:**
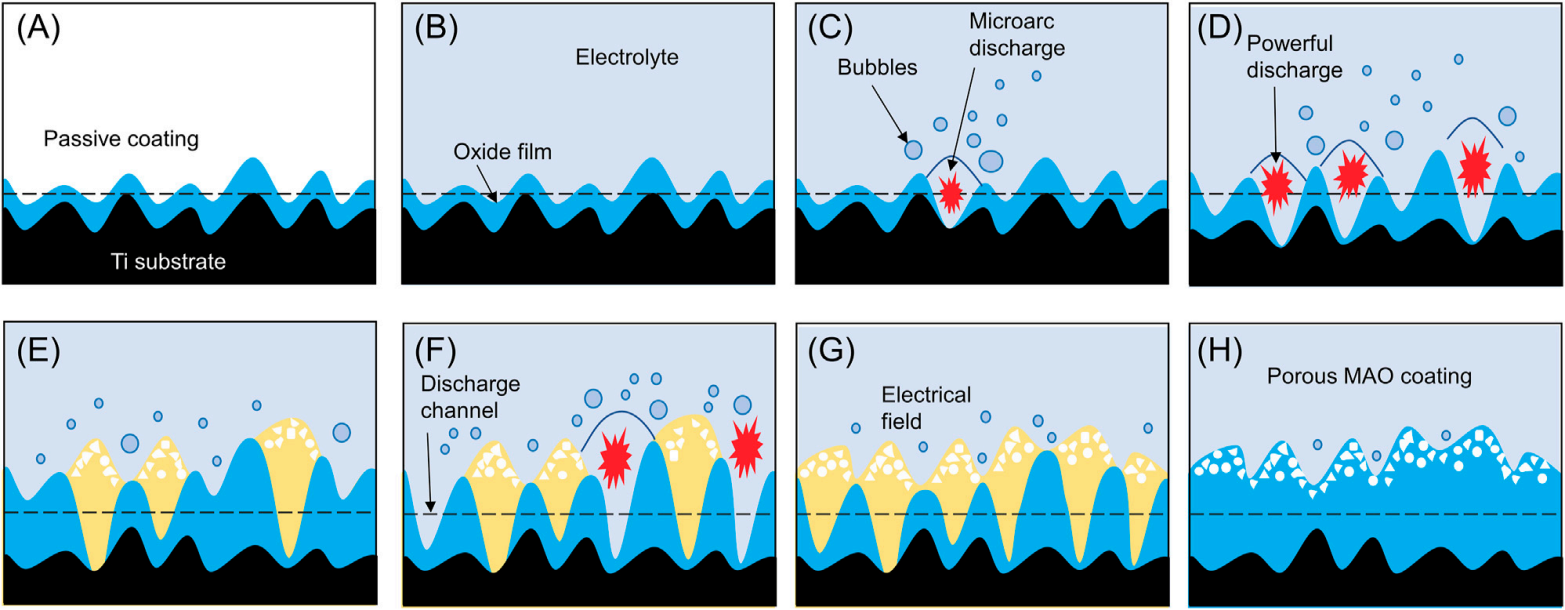
Schematic of the formation process of MAO porous coating. When exposed to air, there will be a layer of passive coating **(A)** on the surface of titanium metal. When the metal is exposed to the electrolyte, a protective film **(B)** will be formed. As the voltage increases, a porous oxide coating **(C,D)** is formed under dielectric breakdown conditions. When the voltage exceeds the dielectric breakdown of the oxide coating, spark discharge will occur, forming larger pores and interconnected microstructure **(E,F)**. The cyclic formation and decomposition of oxide coatings cause potential fluctuations **(G)**, thereby allowing the formation of ceramic oxide coatings by material dissolution and electrolyte gasification **(H)**.

As the metal comes into contact with the electrolyte, a protective film gradually forms. As voltage increases, a porous oxide coating emerges under conditions of dielectric breakdown. When voltage surpasses the dielectric breakdown of the oxide coating, spark discharges occur, forming larger pores and an interconnected microstructure. The cyclic formation and breakdown of the oxide coating cause potential fluctuations, allowing for the formation of a ceramic oxide coating through material dissolution and electrolyte gasification. (Figure 2).

In the case of Ti and its alloys, the formation of the coating during MAO is influenced by various factors, including electrolyte composition, voltage, current density, frequency, reaction time and duty cycle (Chen et al., 2019; Li Z. et al., 2020; Zuo et al., 2020; Huang C. et al., 2021; Yong et al., 2021; Wu and Jiang, 2023). The coating properties obtained after MAO may vary depending on the influence of different parameters. Parameter control aims to obtain a homogeneous, stable, viscous and biologically active surface.

### 2.1 Electrolyte composition

In the MAO process, the choice of electrolyte is of critical significance. The electrolyte not only determines the environment in which MAO occurs but also determines the elemental composition of the final ceramic coating. The electrolyte is important because during the MAO process, the anions in the electrolyte are driven by high voltages, and they strongly bombard the titanium surface, causing them to melt and be deposited in the ceramic coating. Therefore, the chemical composition of the MAO coating depends on the anionic species in the chosen electrolyte. Changing the chemical composition of the electrolyte changes the chemical bonds between the ceramic and titanium, forming the primary bonding force in titanium-ceramic restorations. The electrolyte composition used is established by mixing silicon, calcium, Zn, manganese(Mg) and magnesium particles into the following electrolytes: KOH, Na_2_SiO_3_, NaF, Na_2_P_4_O_7_, and NaAlO_2_ (Pesode and Barve, 2021). To date, the most commonly used electrolytes in MAO technology are Si, P and aluminate-based electrolytes (Walsh et al., 2009; Khorasanian et al., 2011; Shokouhfar et al., 2012). An important factor in the composition of the electrolyte that affects the structure, morphology, and corrosion resistance of MAO coatings on Ti and its alloys is the composition of the electrolyte used (Venkateswarlu et al., 2012; Venkateswarlu et al., 2013; Jiang et al., 2016; Fattah-Alhosseini et al., 2018; Sousa et al., 2021).

Generally, adding silicates to the electrolyte results in thick coatings, rough surfaces and low adhesion. MAO oxide films formed on commercially pure Ti with electrolyte solutions containing silicates have round holes of multiple sizes, whereas P solutions produce mainly round and crater-shaped holes (Shokouhfar et al., 2011; Shokouhfar et al., 2012). In addition, various sodium-based additives in the working electrolyte can significantly affect the corrosion resistance of MAO-modified pure Ti, in terms of microstructure and phase composition (Molaei et al., 2019). Additives such as K2ZrF6 and K2TiF6 in silicate electrolytes can improve the sealed microstructure, increase the hardness and improve the corrosion resistance (Yang et al., 2018). Additionally, wear-resistant ceramic coatings have been generated on novel commercially pure titanium grade 4+ alloys by MAO in an aluminate- and zirconia-containing electrolyte, increasing the hardness and wear resistance of the MAO coatings (Lederer et al., 2021). The research conducted by Xiaohui Yuan et al. shows that using Na2SiO3 or MgSiF6 electrolytes in MAO treatment significantly enhances the bonding strength of titanium-ceramic interfaces. Na_2_SiO_3_ and MgSiF6 groups exhibited uniformly porous MAO coatings, reducing oxygen diffusion and cracks at the titanium-ceramic interface (Yuan et al., 2017).

Phosphate-based electrolytes have low roughness and coating thickness but excellent adhesion. It has been shown that coatings obtained from Ca-P electrolytes exhibit higher surface thickness and corrosion resistance values than coatings obtained from silicate electrolytes (Venkateshwarlu et al., 2012). MAO coatings on valve metals are normally white in appearance, however, colourants, which are normally the salts of transition metal ions, can be used to prepare coloured coatings. Research has shown that MAO treatment can be performed on Ti-6Al-4V alloy in concentrated sodium silicate electrolyte. By controlling the oxidation time, black and white TiO_2_ coatings with silicon dioxide bases are obtained. The short treatment time results in a black coating with excellent corrosion resistance, which is attributed to the presence of Ti^2+^ and Ti^3+^ in the coating. The white coating exhibits good surface roughness and super hydrophilicity. The bond strengths of the black and white coatings on Ti-6Al-4V alloy are approximately 14.4 and 4.3 MPa, respectively (Han et al., 2018). It has been found that MAO treatment of Ti-6Al-4V in a phosphate electrolyte may produce a yellowish colour, while when vanadate is added to the coating, the colour of the coating may change from yellow to brown (Jiang et al., 2016).

A.L. Yerokhin compared the processing and performance of oxide films formed on a Ti-6Al-4V alloy by MAO in aqueous solutions containing aluminate, phosphate, silicate and sulphate anions and certain combinations of these materials. The film produced by the aluminate-phosphate electrolyte is dense and uniform, which was thicker than that produced by the phosphate electrolyte, but both showed good corrosion behaviour. Thicker SiO_2_/TiO_2_-based films with high bulk porosity produced from silicate and silicate-aluminate electrolytes exhibit good corrosion behaviour in H_2_SO_4_ solutions with high chemical stability (Yerokhin et al., 2000).

Hardness is a basic mechanical property of materials. In Gaoqiang Xu’s research, it was found that the hardness of native titanium is low, 265 HV, but the MAO coating on titanium causes a significant increase in the hardness of titanium. It can make the hardness of natural titanium reach 342 HV. After incorporating SiO_2_ nanoparticles, the hardness of MAO-Si increases to 467 HV. The authors concluded that the highest hardness value of MAO-Si can be attributed to the presence of hard SiO_2_ nanoparticles and fewer micropores on the surface of MAO-Si (Xu and Shen, 2019).

### 2.2 Voltage

Voltage variation is another key factor affecting the performance parameters of MAO coatings on Ti and its alloys. Studies have shown that different choices of voltage affect the porosity, pore size, elemental content of the coating, high-temperature resistance, surface morphology, contact angle and surface free energy of the coating (Sedelnikova et al., 2017; Du et al., 2018; Komarova et al., 2020b; Yu et al., 2020). Qing Du’s experimental results show SEM (scanning electron microscopy) images of pure Ti and MAO coatings formed at 300, 350, 400, 450 and 500 V. It was confirmed that a rough and porous surface structure was observed on the MAO-treated Ti plate. A large number of micropores with a size of approximately 1∼3 μm were observed on the MAO coating formed at 300 V. However, as the applied voltage (350∼500 V) increases, the average micropore size on the MAO coating surface increases, while the micropore density decreases. In addition, when the applied voltage is 350–500 V, the uniformity of micropore size decreases. The results show that the applied voltage has an important influence on the surface morphology of MAO coating (Du et al., 2018). Increasing voltage during MAO results in a linear increase in thickness, roughness and porosity of the MAO coatings on Ti and Ti-40Nb substrates, as well as a decrease in adhesion strength values (Komarova et al., 2020a). In addition, Zhao Wang et al. used the MAO technique to fabricate ceramic coatings on AlTiCrVZr alloys and adjusted the voltage to 360, 390, 420 and 450 V during the process (Wang et al., 2023). The resulting coating composition was dominated by Al_2_O_3_, TiO_2_, Cr_2_O_3_, V_2_O_5_, ZrO_2_, and SiO_2_. Additionally, the results showed that the MAO coating gradually became smooth and dense with increasing voltage, the surface roughness decreased the coating thickness increased, and the coating prepared at 420 V showed the best high-temperature oxidation resistance after oxidation for 20 h.

### 2.3 Current

The density, frequency, duty cycle and type of current also have a significant effect on the thickness, surface roughness, corrosion resistance and porosity of MAO coatings on Ti and its alloys (Sobolev et al., 2019; Lederer et al., 2021; Grigoriev et al., 2022; Muntean et al., 2023). For example, Grigoriev et al. discovered that higher current densities increased the coating thickness and surface roughness of Ti-6Al-4V alloys (Grigoriev et al., 2022). Meanwhile, increasing the current density, frequency and duty cycle results in thicker and denser coatings with better tribological properties on pure Ti surfaces (Lederer et al., 2021). Higher current pulse frequencies will produce denser and less porous coatings, resulting in improved corrosion resistance (Sobolev et al., 2019). Finally, for smoother MAO coatings with lower porosity and denser structures on Ti and its alloys, pulsed currents can be used in place of direct currents (Muntean et al., 2023).

Research by Mónica Echeverry-Rendón et al. tested different electrolytes, voltages, current densities and anodization times to obtain surfaces with different properties. The obtained materials were characterized by different techniques such as X-ray diffraction (XRD), SEM and glow discharge emission spectroscopy (GDOES). The results show that compared with the untreated surface, MAO treatment can obtain a super-hydrophilic surface with a contact angle of about 0°, which is hydrophilic, and this situation remains stable after several weeks of MAO in some cases (Echeverry-Rendón et al., 2017).

**FIGURE 3:**
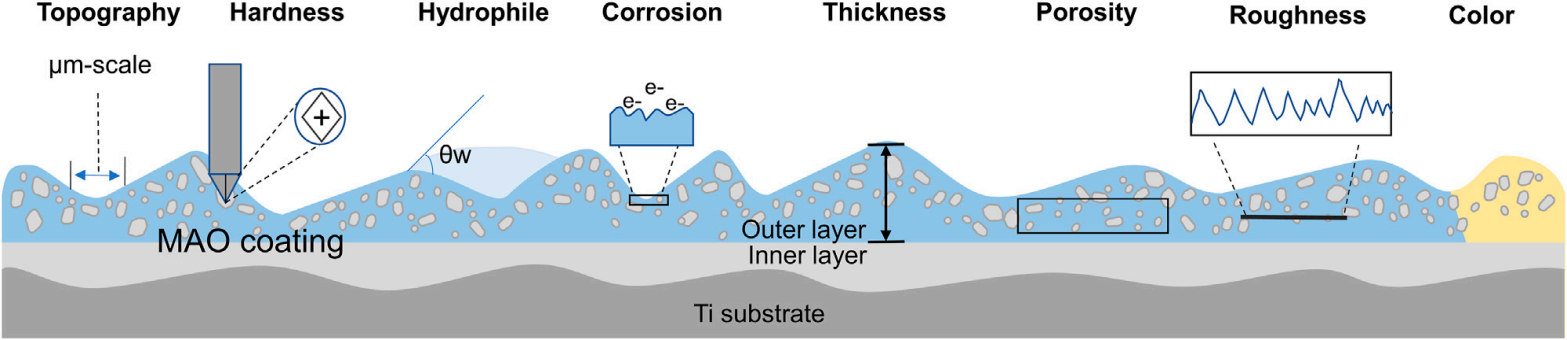
Schematic representation of surface properties modification after MAO deposition.

In summary, the MAO process is a complex technique that requires careful parameter control to achieve desired coating properties. Electrolyte composition, voltage variations and current parameters influence the topography, thickness, roughness, colour, porosity, corrosion resistance, hardness, hydrophilicity, etc., of MAO coatings on Ti and its alloys (Figure 3).

## 3 The biocompatibility of the MAO coating

**FIGURE 4:**
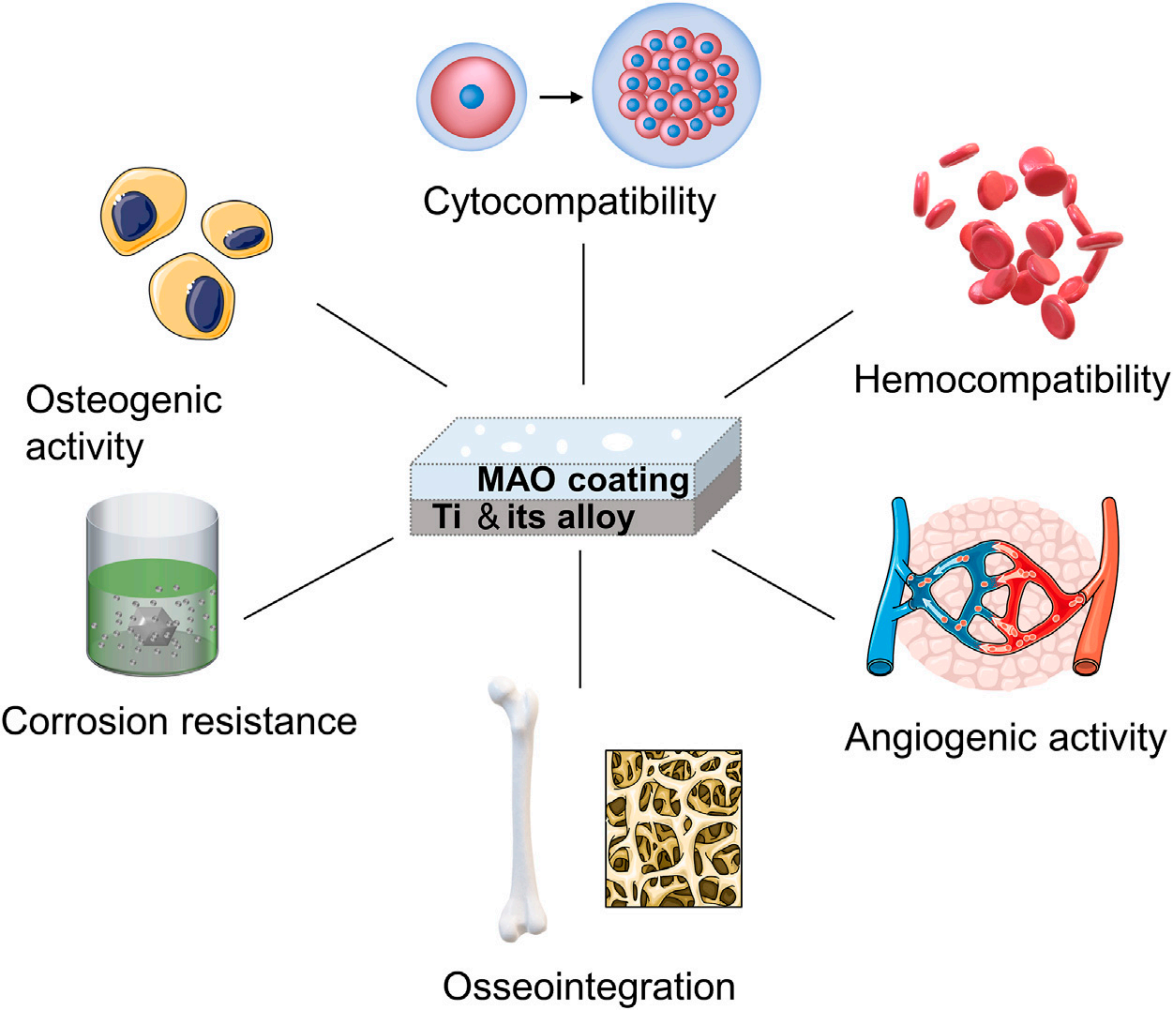
The biocompatibility of the MAO coating.

Biocompatibility is the ability of a material to come into contact with a living organism without causing any adverse or rejection reaction. It is mainly determined by how an organism’s cells, tissues and immune system respond to the material. In the fields of medicine and biology, it is essential to ensure good biocompatibility of materials. MAO technology facilitates the formation of highly biocompatible oxide coatings on Ti and its alloys. Studies on the biocompatibility of MAO coatings on Ti and its alloys focus on cytocompatibility, hemocompatibility, corrosion resistance, angiogenic activity, osteogenic activity and osseointegration (as shown in Figure 4).

### 3.1 Cytocompatibility

Cytocompatibility is the ability of a material to interact with biological cells without causing a toxic or immune response.

**FIGURE 5:**
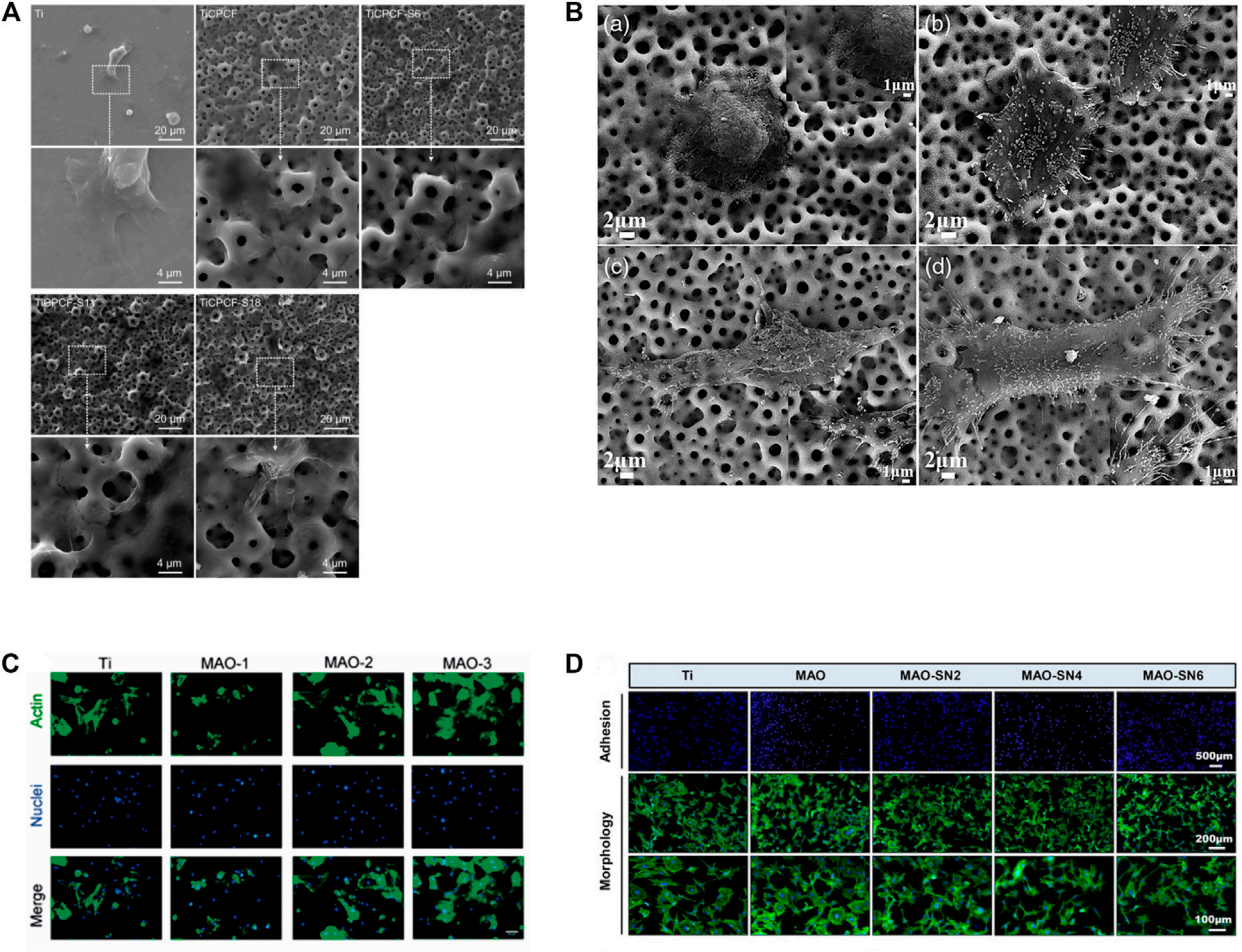
Cell adhesion and extension assay. **(A)** SEM micrographs of MSCs cultured for 3 days. Reprinted from Ref. (Zhou J. et al., 2019). Copyright (2019) Springer Nature. **(B)** SEM images of morphologies of SaOS-2 cells adhered to the MAO: A 4 h and C 24 h; MHTZn: B 4 h and D 24 h. Adapted from Ref. (Zhang et al., 2019). ^©^ 2019 The Author(s). Published by Informa UK Limited, trading as Taylor & Francis Group. **(C)** BMSCs were stained by FITC-phalloidin and DAPI after incubation for 4 h on four samples. Scale bar = 50 mm. Reprinted from Ref. (Zhou W. et al., 2019). Copyright (2019) Taylor & Francis. **(D)** Representative staining images of the early adhesion and morphology of MC3T3-E1 cells. Reprinted from Ref. (Shen Y. et al., 2022). Copyright (2022) Frontiers.

Various cell types have been shown to adhere and proliferate better on MAO-modified Ti and its alloys, including bone marrow stromal stem cells (BMSCs), MC3T3-E1 cells, MG63 cell lines, hMSCs, NIH-3T3, immortalized skin fibroblasts, rabbit mesenchymal stem cells and endothelial cells (Xiu et al., 2016; Zhang X. et al., 2018; Zhou W. et al., 2019; Zhou et al., 2019 J.; Liao et al., 2020; Shen Y. et al., 2022; Kostelac et al., 2022; Molaei et al., 2022). MAO-treated specimens have highly porous layers that can be observed under SEM. Cells on MAO exhibit polygonal shapes with filamentous and lamellar extensions of pseudopods, improved spreading, increased cell density and pseudopods anchored in micropores (Zhou J. et al., 2019; Pan et al., 2019; Zhang et al., 2019; Li Y. et al., 2020; Kostelac et al., 2022; Wang et al., 2022). The degrees of cell adhesion and proliferation on Ti and its alloy MAO coatings can be influenced by adding different elements or by adjusting the ion concentration in the electrolyte (Wang L. J. et al., 2021; Shen Y. et al., 2022). The longer the MAO process is, the larger the pore size on the coating and the more cells are attached (Zhou W. et al., 2019). Cell adhesion and extension assay on MAO coating on Ti and its alloy as shown in (Figure 5) (Zhou J. et al., 2019; Zhou W. et al., 2019; Zhang et al., 2019; Shen Y. et al., 2022). In addition, under an SEM electron microscope, the smooth titanium surface has parallel scratches arranged along the grinding direction. The surface of the coating treated by micro-arc oxidation shows a typical nano-porous structure. As the duty cycle increases, the surface of the coating shows micron-sized grooves and nano-sized pores that are evenly distributed and connected. Cell proliferation is increasingly obvious in structures with nanoscale porous and micron-scale grooves. (Pan et al., 2019).

Scholars have shown that the MAO-treated Ti surface creates a safe coating that reduces the cytotoxicity of the metal. For example, Ti and its alloys covered with MAO coatings containing Mg, Ca, P, Sr, Co and F show no cellular toxicity, differing from untreated Ti (Zhou J. et al., 2019; Li X. et al., 2020). Additionally, Lorena Kostelac conducted cell adhesion tests to confirm the cytocompatibility of the hydroxyapatite (HAP) coating obtained on the surface of titanium alloys by MAO. Human skin fibroblasts are first incubated with the samples for 48 h and then stained with calcein-AM to visualize viable cells on titanium substrates. The results showed that after 2 days of cell culture, no cytotoxic effects were observed, and fibroblasts spread and colonized all available areas on the sample surface. Furthermore, higher cell densities are observed in all MAO-treated substrates relative to untreated substrates. (Kostelac et al., 2022). However, it is important to note that the presence of excess Cu ions in the coating inhibits cellular activity (Zhang X. et al., 2018). There are also high and low levels of cytotoxicity for each coating, with one study finding that Ti-Ag2O coatings are less cytotoxic than Ti-Ag coatings (Zhang et al., 2021c).

In the design of multifunctional surface coatings for titanium and its alloys, the nanoscale is sometimes involved. Nanoparticles (NPs) can bind to antibodies, ligands, and drugs, thereby enhancing their potential applicability in biotechnology, drug and gene delivery, magnetic separation, and imaging; in addition to their favour properties, they can cause harmful effects if they enter living biological systems and tissues (Attarilar et al., 2020). The formation of MAO coatings on Ti-6Al-4V alloy surfaces with ZrO_2_ and ZnO nanoparticles significantly improves the corrosion resistance of MAO-treated Ti and its alloys, with corrosion rates that are approximately 14 times lower than those of the substrate (Nadimi and Dehghanian, 2021). These results suggest that MAO treatment is beneficial in reducing cytotoxicity.

Therefore, MAO treatment can create a safe coating on the surface of Ti and its alloys to enhance the cytocompatibility of Ti and its alloys to promote cell adhesion, proliferation and differentiation.

### 3.2 Hemocompatibility

Hemocompatibility refers to the biological reactions and effects that occur when a material comes into contact with blood. One of the important indicators to examine the hemocompatibility of blood contact materials is the hemolysis rate. Studies have shown that the hemolysis rates of MAO-treated Ti-6Al-4V alloy and untreated Ti-6Al-4V alloy are 0.85% and 1.51% respectively, indicating that the former has better hemocompatibility. Furthermore, MAO treatment reduces platelet adhesion and activation to the surface of Ti and its alloys which exhibit a uniform but rough porous microstructure (crater-like), inhibits thrombosis and inflammatory responses, and ultimately reduces the risk of vascular endothelial cell damage (Jiang et al., 2015). However, it should be noted that not all MAO treatments improve hemocompatibility and, in some cases, may even reduce haemotolerance. For example, it has been shown that platelet coverage on MAO-treated Ti surfaces can be higher than that without MAO treatment (Klein et al., 2020). Wang Maosheng et al. also found that platelets spread on MAO-treated surfaces and exhibited extensive pseudopods. The interaction between platelets and the MAO-treated surface resulted in the formation of an almost connected platelet layer (Wang M.-S. et al., 2015).

### 3.3 Corrosion resistance

To be able to evaluate the quality of the coatings produced by MAO on Ti and its alloys, it is necessary to test their corrosion resistance, particularly regarding evaluating their biocompatibility (Molaeipour et al., 2022). For biomedical applications, simulated body fluids (SBF) are often used as electrolytes because of their ionic composition, which is similar to that of human blood plasma. Studies have shown that pure Ti with MAO coating immersed in SBF exhibited no significant damage or cracking, while untreated CP-Ti showed ulcerative corrosion (Molaei et al., 2022). In addition, the corrosion current density of the Ti alloy samples with MAO coating decreased by more than 15 times in SBF. A comparative analysis of the corrosion behaviour of samples with and without protective coating in SBF was performed. Potentiodynamic polarization data reveals an improvement in corrosion properties after surface coating with an MAO layer (Mashtalyar et al., 2020). Likewise, a study by Gaoqiang Xu showed that the MAO technique can form wear and corrosion-resistant coatings on Ti and its alloys (Xu and Shen, 2019). These results suggest that in the human body, MAO-treated Ti and its alloy implants may possess good corrosion resistance.

### 3.4 Angiogenic activity

Studies have demonstrated that the application of MAO coatings onto the surface of Ti and its alloys can augment the attachment, multiplication, differentiation, and transmigration of vascular endothelial cells and fibroblasts. This ultimately results in invigorating tissue repair and regeneration by inspiring vascular rejuvenation. For instance, research by Yiding Shen et al. revealed that MAO-modified titanium evinced noteworthy enhancement in the viability and translocation of human umbilical vein endothelial cells when compared to untreated titanium. Cells on the surface of MAO-treated titanium and its alloys exhibited intricate vascular networks and significantly high levels of vascular genes, indicating the potential for rapid angiogenesis *in vitro* (Shen Y. et al., 2022). Additionally, mesenchymal stem cells co-cultured with MAO coatings witnessed an elevation in angiogenic factors like HIF-1a and VEGF (Zhou J. et al., 2019). Adding Cu NPs to TiO_2_ coatings was also found to amplify endothelial cell proliferation and VEGF secretion (Zhang X. et al., 2018). In conclusion, these findings suggest that MAO-treated Ti and its alloys have immense potential for enhancing tissue repair and regeneration by stimulating angiogenesis.

### 3.5 Osteogenic activity

Osteogenic activity refers to the ability of a material to promote the proliferation and differentiation of bone cells, thereby accelerating the repair and regeneration of bone tissue. The MAO coating on the surface of Ti and its alloys, with its porous structure, have been found to improve the adhesion and bioactivity of osteoblasts while releasing beneficial ions and molecules that stimulate the proliferation and differentiation of osteoblasts and the formation of new bone tissue. Various studies have shown that MAO coatings can increase extracellular matrix mineralization and bone-like apatite deposition by osteoblasts, indicating desirable osteogenic activity (Zhang X. et al., 2018; Zhang. et al., 2021c; Shen Y. et al., 2022). In addition, MAO treatment has been found to increase alkaline phosphatase (ALP) activity, along with a wider positive area for ALP staining than the untreated group (Zhou W. et al., 2019; Zhao et al., 2020). The expression of genes related to osteogenesis is also critical in determining osteoblast activity. qRT-PCR studies have shown that MAO treatment significantly increased the expression levels of osteogenic genes such as Runx2, ALP and osteocalcin (OCN) in Ti and its alloys compared to the untreated group (Wang et al., 2022).

Furthermore, the effect of MAO coating on osteoblasts plays an important role in determining osteoblast activity. Studies have shown that MAO-coated Ti and its alloys promote the spread and growth of osteoclasts on their surface and increase osteoclasts (Santos-Coquillat et al., 2019).

Inappropriate use of antibiotics leads to the proliferation of drug-resistant bacteria and the emergence of “superbugs,” such as methicillin-resistant *Staphylococcus aureus* (*MRSA*), Multidrug-resistant (MDR) *E. coli* (*Escherichia coli*), Multidrug-resistant Pneumonia *Klebsiella*, and vancomycin-resistant *Enterococcus* (VRE). (Xia et al., 2023). The misuse of antibiotics has increased in cases of osteomyelitis. In the study conducted by Teng Zhang and others, they developed a novel multilevel structured MAO 3D-printed porous Ti6Al4V scaffold for the sustained release of vancomycin. The scholars designed polydopamine (PDA) as an adhesive anchor for heparin; the adhesive was affixed to the underlying microporous MAO-TiO_2_/CaP layers. As confirmed by high-performance liquid chromatography, this system demonstrates high loading capacity and sustained vancomycin release kinetics. *In vivo* experiments, they injected 0.1 mL of 108 colony-forming units (CFU) of MRSA into the tibiae of rabbits to induce severe osteomyelitis. Physical, haematological, radiological, microbiological, and histopathological analyses were conducted to assess the therapeutic effects. It has been found that rabbits treated with vancomycin-loaded MAO scaffolds inhibit bone infection and enhance bone formation. (Zhang et al., 2020).

### 3.6 Osseointegration

Osseointegration is an important aspect of ensuring the long-term stability and function of artificial implants in the host bone tissue. It shows that the MAO coating process promotes osseointegration by creating a highly porous and rough surface that improves interfacial bonding between bone tissue and Ti and its alloy implants (Huang L. et al., 2021).

**FIGURE 6:**
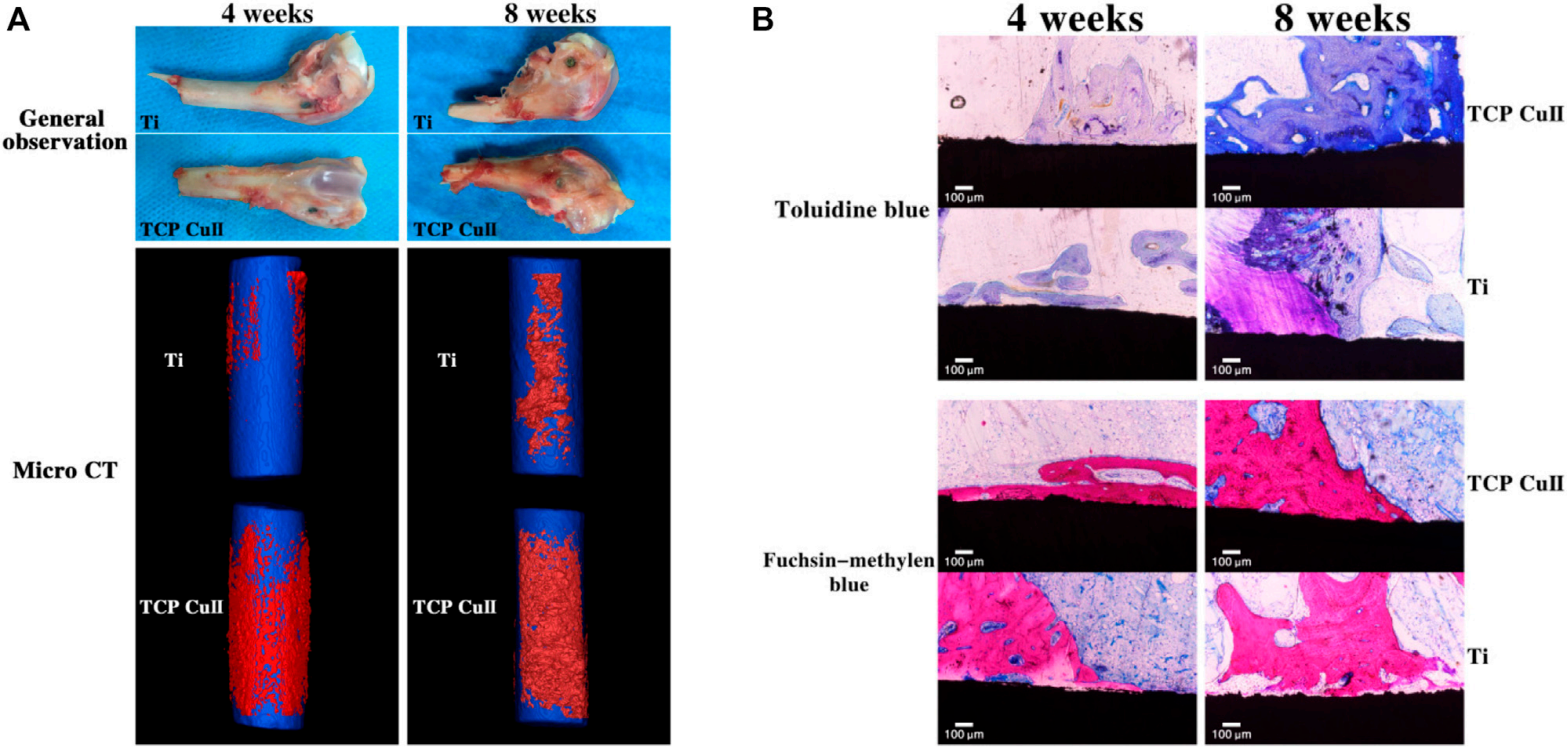
Osseointegration assay. **(A)** Gross observation and micro-CT reconstruction of the femoral condyle were observed at 4 and 8 weeks after implantation. **(B)** Toluidine blue and fuchsin-methylene blue staining of new bone formation at 4 and 8 weeks after implantation. Reprinted from Ref. (Wang L. J. et al., 2021). Copyright (2021) Springer Nature.

MAO-treated Ti-6Al-4V alloys can promote bone growth and implantation in healthy adult male New Zealand rabbits, with early osteogenesis occurring primarily on the implant surface, followed by external expansion (Xiu et al., 2016). Histological examination showed that the microporous clocks of MAO-treated Ti had more bone tissue surrounded by a thicker and continuous bone matrix than untreated Ti, demonstrating the effectiveness of the MAO coating process in promoting osseointegration (Xiu et al., 2016). Similar experiments have shown that 8 weeks after the placement of Ti implants in adult rabbits, the pure Ti surface was still covered with fibrous tissue, while new bone formed on the surface of the titanium implants with MAO coating (Zhao et al., 2020). Other experiments have shown that MAO-coated implants have better bone-to-implant contact and coverage area attached to the implant, the highest density of newly grown paragenetic bone tissue, and can withstand greater shear (Chen et al., 2021; Ding et al., 2022; Ni et al., 2022). Wang et al.'s study demonstrated good osseointegration of the MAO coating was found by observing gross observation and micro-CT reconstruction of the femoral condyle after implant implantation and new bone formation was observed by toluidine blue and fuchsia-methylene blue staining. (Figure 6); (Wang L. J. et al., 2021). Compared to smooth surfaces, the MAO-treated group had more aggressive contact and much distant osteogenesis (Li et al., 2018).

Strontium (Sr), as an essential microelement that acts similarly to calcium with potent bone-seeking properties, has been proven to be an integral feature in the development of bone and/or teeth. Sr-doped implants have been proven to significantly promote early osseointegration under normal and osteoporotic conditions. (Lu W. et al., 2022). It has been reported that Sr prevents osteoporosis via two mechanisms, simultaneously stimulating osteogenesis and restraining osteoclastogenesis via diverse cellular signalling pathways. Recently, Zhou et al. discovered that Sr-doped titanium samples can significantly promote osteogenesis by decreasing ROS expression and inhibiting adipogenic differentiation of mesenchymal stem cells in ageing rats. (Zhou C. et al., 2019). In addition, scientists have implanted MAO coatings into osteoporotic rats and found that high concentrations of Sr^2+^ significantly reduce the expression of ROS in osteoporosis, showing good results (Shen X. et al., 2022). Overall, MAO significantly improves fixation strength, bone formation, and osseointegration with the Ti and its alloy implant surface.

HA is a biologically active material widely used to enhance the osseointegration of titanium dental implants. During the MAO process, an electrolyte solution containing calcium and phosphate ions is utilized, which can result in the formation of HA within the oxide layer. Subsequent hydrothermal treatment can be employed to increase the HA content. This phenomenon in a titanium oxide surface with high porosity, controlled thickness, and a considerable density of HA. (Lugovskoy and Lugovskoy, 2014).

According to research in the literature, the utilization of HA coatings with a nanoparticle structure can facilitate the adhesion, spreading, and differentiation of bone cells (MC3T3-E1), leading to an improvement in their overall activity. It should be noted, however, that excessive deposition of a large quantity of nano-HA particles on the surface may have adverse effects on cell proliferation (Wang X. et al., 2021)**.**

Additionally, by applying MAO treatment and microwave hydrothermal treatment, a coating containing HA crystals can be rapidly formed on the titanium surface. Research results show that HA can dissolve in SBF, thus increasing the local supersaturation of Ca and phosphorus(P) elements near the coating surface. Furthermore, the material exhibits a good crystallographic match with the deposited apatite, accelerating the formation of the deposited apatite layer. Therefore, this surface demonstrates good cell activity and osseointegration capabilities due to its favourable wettability, high surface energy, and aptitude for apatite formation. (Du et al., 2019).

In summary, the MAO technique can form a layer of biocompatible coating on the surface of Ti and its alloys. This coating promotes the proliferation, adhesion and differentiation of many cell types on the surface and has high cytocompatibility and haem compatibility. In addition, this coating has excellent corrosion resistance and remains stable during long-term use. Importantly, this coating can promote bone formation and therefore has broad application prospects in the medical field.

## 4 The antibacterial properties of the MAO coating

**FIGURE 7:**
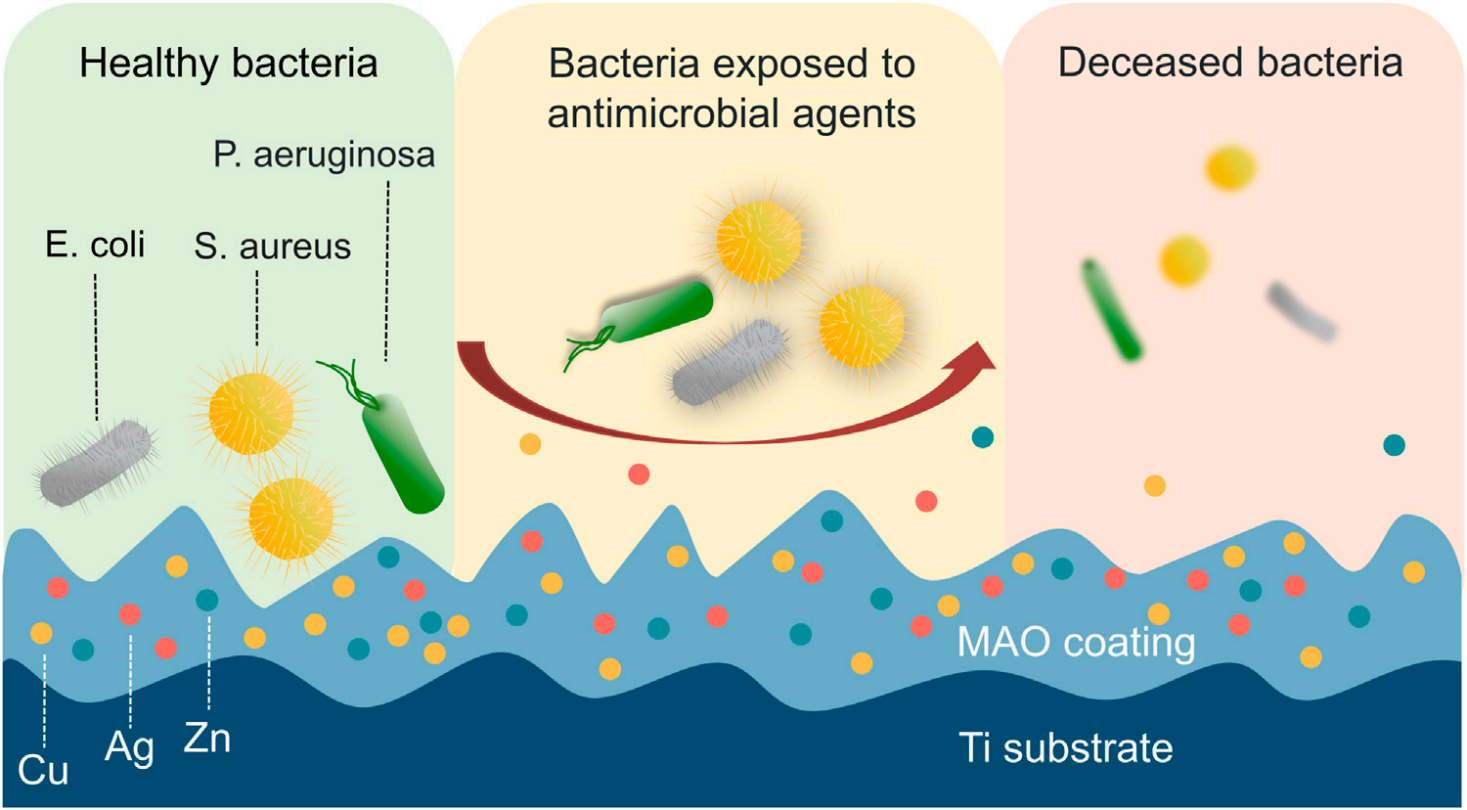
Direct contact with antimicrobial elements causes physical damage and cell death in bacteria. The bacteria can experience physical damage to their membrane upon direct contact with antimicrobial elements, including Cu, Ag, and Zn, which ultimately leads to cell death.

Bacterial infection remains a significant global threat worldwide owing to its potential to cause multiple organ damage and increase the risk of other diseases. (Tang et al., 2021; Huang et al., 2023; Ji et al., 2023; Lin et al., 2023). Similarly, the use of MAO-treated materials for medical applications is also often hampered by the potential risk of bacterial infection. To address this issue, researchers have explored the use of metallic elements such as Cu, Ag, Zn, Mg, etc., as well as biological antimicrobial agents to enhance the antimicrobial properties of MAO coatings on Ti and its alloys. (Figure 7). Incorporating these elements into the coating can help reduce the risk of bacterial infection, making MAO-treated materials more suitable for medical applications (Supplementary Table S1).

### 4.1 Cu-containing MAO coating

Cu is a vital trace element in the human body with excellent antibacterial properties (Gross et al., 2019). Studies have shown that Cu can enhance lipid peroxidation, inhibit bacterial active DNA and related enzymes, interfere with bacterial energy metabolism, and exhibit a low potential for drug resistance (Shimabukuro, 2020). In addition, Cu ions can kill bacteria or disrupt their replication using direct contact and ion release (Huang et al., 2018; Wang et al., 2019a; He et al., 2020).

**FIGURE 8:**
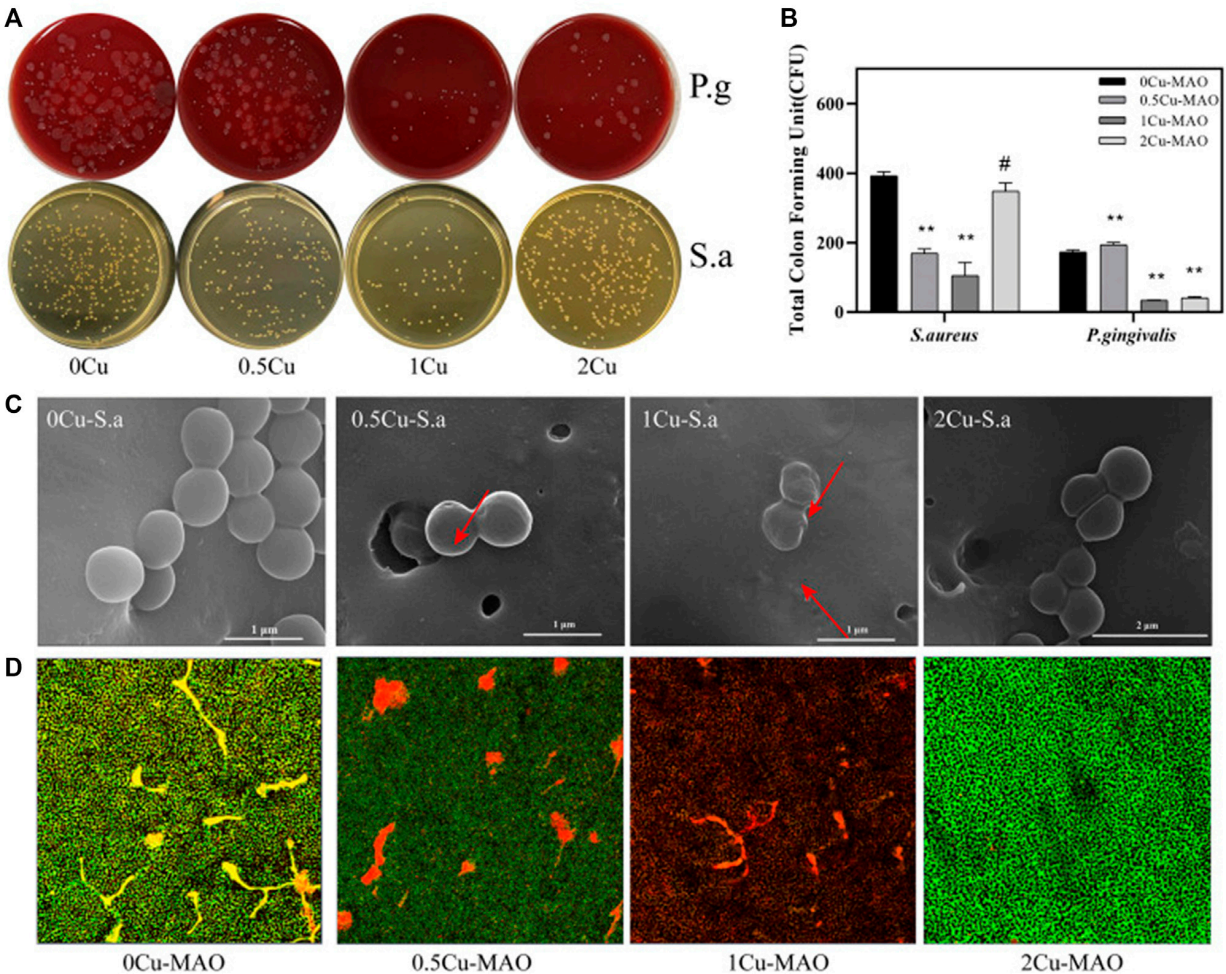
Antibacterial activities of Cu-doped coatings against *S. aureus* and P. gingivalis **(A)**; CFU results after *S. aureus* and P. gingivalis were cocultured with the Cu-MAO coating for 24 h. Data are presented as mean ± SD; (**p < 0.01, vs. 0Cu-MAO group); **(B)**; Live/Dead assay **(D)** and the corresponding SEM images **(C)** of *S. aureus* cultured on different surface after 24 h of incubation. Source: Reprinted from Ref. (Kang et al., 2022). Copyright ^©^ 2022 kang, Lan, Yao, Liu, Chen and Qi, with permission from Frontiers in Bioengineering and Biotechnology.

The MAO process can be used to successfully produce Cu-containing coatings on Ti and its alloy, resulting in substantially antimicrobial properties (Lu X. et al., 2022). In a related study by Binbin Kang, Cu doping has been found to effectively promote the proliferation of BMSCs and enhance osteogenic differentiation when compared to TiO_2_ coating. Furthermore, the antibacterial experiments conducted revealed that Cu-doped TiO_2_ coating had a significant impact on *Streptococcus mutans* (*S. aureus*) and *Porphyromonas gingivalis* (*P. gingivalis*), demonstrating exceptional antibacterial properties (Figure 8) (Kang et al., 2022).

Furthermore, studies have shown that Cu ions exhibit high bioactivity and excellent antibacterial properties over a range of concentrations and that higher concentrations of Cu lead to better antibacterial effects. The research findings indicate that all samples exhibit morphological features indicative of sedimentation phenomena, with porosity following descending order: Ti−10Cu (∼1.87%) > Ti−5Cu (∼1.55%) > Ti−2Cu (∼0.82%). In addition to the presence of flocculent deposits, the Ti−2Cu coating also exhibits spherical, rod-like, and lamellar deposits. Similarly, typical micron-scale clusters containing sediment of various morphologies are observed in the Ti−5Cu coating. Conversely, the Ti−10Cu coating exclusively features lamellar deposits. The antimicrobial capability of the Ti−2Cu, Ti−5Cu, and Ti−10Cu coatings was assessed by measuring the OD600 value of the medium after 24 h of incubation. The results showed that the OD600 value of the copper-doped TiO2 coating was significantly reduced compared with the titanium substrate, indicating that the copper-doped was beneficial to its antibacterial effect. However, the antibacterial efficacy is inconsistent with its Cu content, because the Ti−2Cu coating shows higher antibacterial efficacy than the Ti−5Cu coating, and the Ti−5Cu coating is similar to the Ti−10Cu coating (Zhang X. et al., 2018).

The studies show that the antimicrobial concentrations of Cu ranging from 0.54% to 0.72% on the surface of MAO coatings showed antibacterial and fungicidal properties (Zheng et al., 2018; Rokosz et al., 2020). However, Zhang Xinxin et al. found that the antibacterial rate (AR) of the TiO2 coating formed by adding 2 g/L Na_2_Cu EDTA was similar to that of adding 10 g/L; both were superior to the 5 g/L coating (Zhang et al., 2021b). Therefore, the modulation of Cu concentration needs to be further investigated.

Additionally, aside from Cu concentration, the antimicrobial properties of MAO coatings on Ti and its alloys are influenced by the price of Cu ions. It has been shown that Cu-doped TiO_2_ coatings in the Cu^2+^ enriched region have better antimicrobial activity compared to those with high Cu + content (Zhang et al., 2020e). Furthermore, t increasing the Cu+/Cu2+ content of the coating leads to better antibacterial and antifungal effects (Rokosz et al., 2020).

MAO coatings with Cu ions have been found to possess powerful long-term antibacterial properties. For example, a porous three-dimensional (3D) MAO coating containing 1.92 wt% Cu (Cu-1.92 wt%) developed on a Ti-6Al-4V alloy maintained 100% antimicrobial activity against *S. aureus* (*S. aureus*) even after being immersed in a Hanks solution for 14 days (Liang et al., 2020). In addition, studies have shown Cu-based MAO coatings on Ti alloys exhibit exceptional antibacterial activity against Gram-positive and Gram-negative bacteria even after being submerged in normal brine for 28 days (Shimabukuro et al., 2020b).

The Cu-containing MAO coating achieved 98.7 antibacterial activity against *S. mutans*, effectively killing *S. mutans* on almost all surfaces, and also demonstrated potent antibacterial effects against *S. aureus* and *E. coli* (Shen et al., 2020a). These results indicate that Cu-based MAO coatings on Ti and its alloys have strong antibacterial properties.

### 4.2 Ag-containing MAO coating

Ag is a prevalent metal known for its antibacterial properties. The presence of Ag ions can disrupt the membrane integrity of bacterial cells, leading to their death. Many studies have demonstrated that Ag-containing TiO2 coatings can impede the proliferation and adhesion of bacteria such as *S. aureus* while showing extremely high bactericidal activity against multiple antibacterial strains of *S. aureus* (e.g., USA300), *E. coli* and *Pseudomonas aeruginosa* (*P. aeruginosa*), which can destroy planktonic and adherent bacteria (Sedelnikova et al., 2019; Zhang L. et al., 2020; Zhang et al., 2021c; van Hengel et al., 2020; Zhao et al., 2022).

Through SEM analysis, it was observed the Ag-MAO coating was uniformly covered with micro/nanoporous oxide layers. Furthermore, the surface morphology of the biofunctionalized implants remained unaltered by the addition of AgNPs when compared to pure titanium and pure titanium combined with strontium implants. Results indicated that after 24 h, Ag-MAO coating completely prevented bacteria from adhering to the surface. After 48 h, little to no bacteria was observed on the surface of the Ag-MAO samples, whereas the control group had significant bacterial adhesion. *In vivo* experiments on murine femurs showed that the Ag-MAO samples were effective in eliminating bacterial inoculum (Thukkaram et al., 2020). Besides, it has been found that higher concentrations of AgNPs or Ag2O nanoparticles in the electrolyte yield better antibacterial effects for Ag-doped TiO2 coatings. (Lv et al., 2019; Thukkaram et al., 2020). Moreover, TiO2 coatings containing 18.5 wt% Sr and 0.58 wt% Ag showed good osteogenic activity on MC3T3-E1 cells and strong short- and long-term antibacterial effects (Zhang Y.-Y. et al., 2021).

While Ag-rich MAO coatings are highly biocompatible with U2OS cell lines in some studies (Oleshko et al., 2020b), it is important to note that excessive amounts of Ag content can reduce the cytocompatibility of the coating. For instance, coatings synthesised with a concentration of ≥0.001 mol L^−1^ AgC_2_H_3_O_2_ to the electrolyte have been shown to harm the proliferation of Saos-2 cells (Teker Aydogan et al., 2018). In their research, Masaya Shimabukuro et al. found that MAO coatings of Ti prepared with an electrolyte containing Ag nitrate at a concentration of 0.04 mm or higher exhibited good antibacterial effects, but when Ag concentration exceeded 2.5 mm, the resulting samples inhibited the activity of MC3T3-E1 cells (Shimabukuro et al., 2019). Therefore, it is crucial to pay attention to the concentration of Ag when using it in the MAO treatment of Ti and its alloys, selecting concentrations that are highly effective against bacteria while still exhibiting biocompatibility.

### 4.3 Zn-containing MAO coating

Zn is an essential trace element for the human body and an important component of superoxide dismutase (Siddiqi et al., 2018). Studies have shown that the incorporation of Zn onto the surface of Ti and its alloys has been shown to not only act as an effective antimicrobial agent but also improve cytocompatibility (Hu et al., 2022; Qin et al., 2022). It was reported that TiO2 coatings significantly inhibited bacterial growth without causing cytotoxicity at concentrations of Zn ions between 10^−5^ and 10^−4^ M (Zhang et al., 2016).

In addition, Zn was doped on the surface of Ti-15Mo MAO coating, which was found to be effective in reducing the number of attached *Staphylococcus* epidermidis (ATCC 700296), indicating that Zn can prevent the formation of bacterial biofilm on the implant surface (Leśniak-Ziółkowska et al., 2020). Oleksandr Oleshko et al. used MAO technology to combine ZnO nanoparticles (NPS) with TiZrNb alloys to form high-contact angle ceramic coatings on TiZrNb alloys. Observe the sample with SEM, the surface is covered with an oxide layer, containing small circular holes and craters of various sizes. This coating containing ZnO NP prevents the adhesion of *S. aureus* and significantly improves the antimicrobial properties of TiZrNb alloys (Oleshko et al., 2020a).

### 4.4 Multiple elements-containing MAO coating

Recent research has shown that incorporating multiple elements into MAO coatings can lead to a stronger antibacterial effect than using a single metal element. For instance, TiO_2_ coatings mixed with both Ag and Zn showed higher antibacterial activity against *S. aureus* than Ti substrates containing only Ag or Zn alone (Lv et al., 2020; Lv et al., 2021b). Cu, Zn, and P were doped into Ti-6Al-4V Ti alloy coatings via MAO, which showed good antibacterial properties against *E. coli*, *S. aureus*, and MRSA (Wang et al., 2020). Meanwhile, TiO_2_ coatings doped with Mg, Cu, and fluoride (F) elements on the Ti surface were more effective in inhibiting *S. aureus* than pure Ti and oxide coatings containing Mg, Cu, or F alone (Zhao et al., 2019). In addition, doping multiple metal elements into the coatings not only improved the antibacterial properties of Ti and its alloys but also enhanced biocompatibility. For example, co-doping 0.55 wt% Cu and 2.53 wt% Zn further boosted the proliferation of L-929 cells compared to TiO_2_ doped with 0.77 wt% Cu alone (Zhang et al., 2018a).

Recent studies have revealed that additional elements beyond the usual Cu, Ag, and Zn elements can be added to MAO coatings to confer antibacterial effects on Ti and its alloys. Elements like Zirconium (Zr), Boron (B), Manganese (Mn), Tungsten (W), Yttrium (Y), and Fluorine (F), have demonstrated bactericidal effects against *S. aureus*, *P. aeruginosa*, and *E. coli* (Sopchenski et al., 2018; Zhou J. et al., 2019; Zhou et al., 2019 T.; Zhang et al., 2020d; Zhang et al., 2020a; Nikoomanzari et al., 2020; Molaei et al., 2022).

In studies evaluating MAO coatings on Ti and its alloys for antibacterial, scholars have also utilized electrolytes containing bioactive elements like Na_2_SiO_3_-5H_2_O, C_4_H_6_O_4_Ca, NaNO_3_, and C_3_H_7_Na_2_O_6_P to form bioactive glass-based coatings (MAO-BG). MAO-BG coatings were found to reduce pathogenic bacteria associated with biofilms and have positive effects on various microbial biofilms. This suggests that incorporating bioactive elements into Ti materials could be a useful way to impart antimicrobial properties (Costa et al., 2020).

To summarize, the incorporation of multiple elements into MAO coatings has the potential to enhance the antimicrobial properties and biocompatibility of Ti and its alloys. In addition to commonly used elements like Cu, Ag, and Zn, alternative elements like Zr, B, Mn, W, Y, and F have shown notable bactericidal effects in MAO coatings. Moreover, MAO-BG coatings formed by electrolytes containing bioactive elements can effectively impart antimicrobial properties to Ti materials and positively impact different microbial biofilms. These discoveries offer novel approaches and concepts for developing more powerful antimicrobial coatings.

## 5 Combined application of MAO and other treatment methods

**FIGURE 9:**
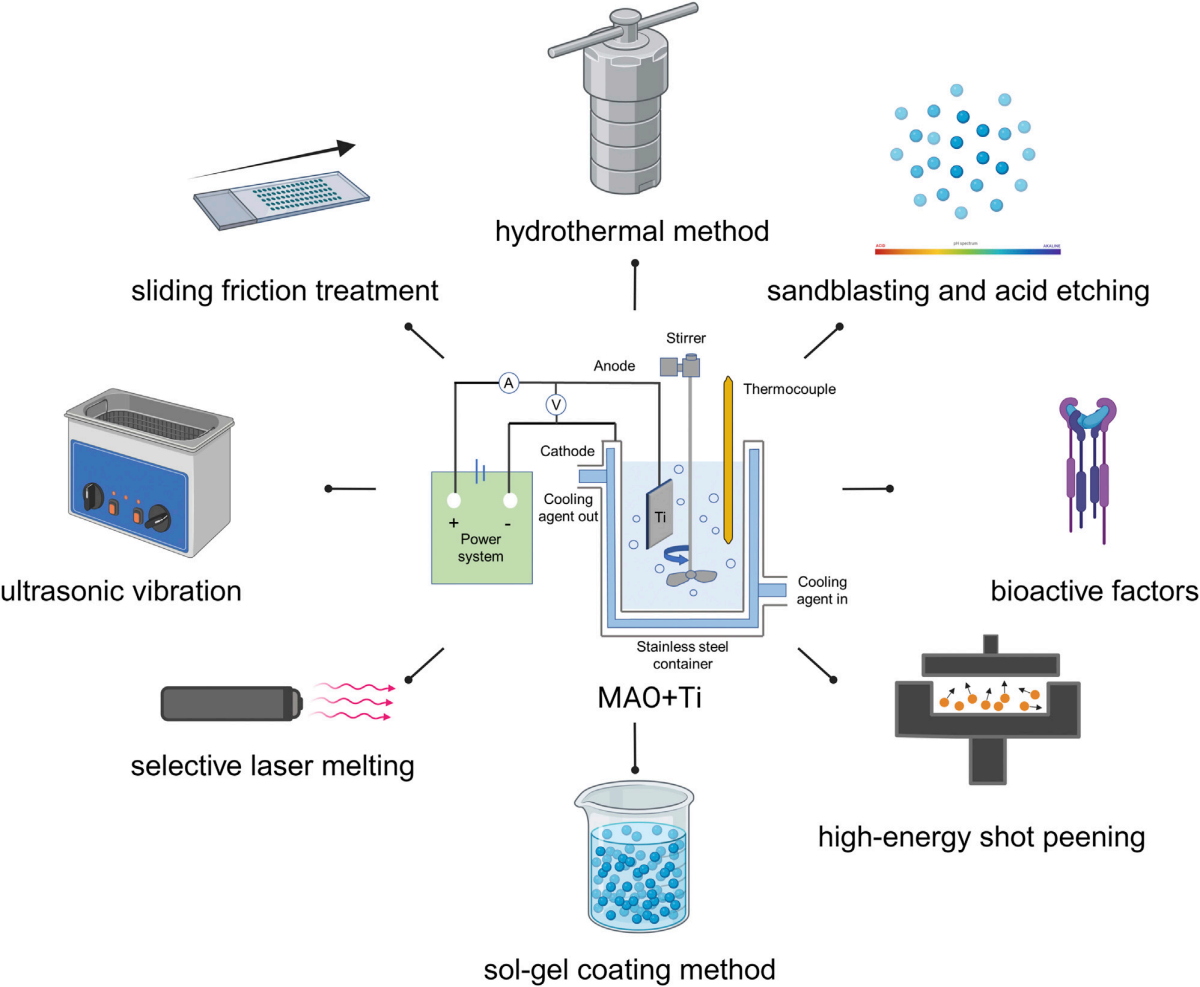
Combined application of MAO and other treatment methods. Created with BioRender.com.

In modern times, it has been discovered that relying solely on MAO technology may not always suffice for certain biomedical surface modification needs. As a result, researchers have delved into exploring the use of MAO combination with other surface treatment methods, including but not limited to hydrothermal method, sandblasting and acid etching (SLA), selective laser melting (SLM), high-energy shot peening (HESP), sliding friction treatment (SFT), sol-gel coating method (SG), ultrasonic vibration (UV), and bioactive factors (Figure 9). By utilizing a combination of these methods, researchers aim to achieve the desired level of effect (as outlined in Table 2).

**TABLE 2:** Combined application of MAO with other treatment methods.

Treatments	Substrate	Surface morphology	Cell models	Animal models	Bacteria	Outcome	Reference
MAO and hydrothermal treatment	Ti6Al4V	Micro-nano hybrid coating with moderate roughness	MC3T3-E1 cells	rat	-	Enhance biocompatibility, osteogenesis, and osseointegration	Huang et al. (2021)
MAO and microwave hydrothermal treatment	Ti	Slightly higher sub-micron roughness	BMMSC			Enhancing the differentiation of osteoprogenitor stem cell	Lin et al. (2020)
MAO and Steam-Hydrothermal treatment	Ti	Volcanic porous structure and nano-structures	MC3T3-E1 cells, human umbilical vein fusion cells	rabbits	-	Promote Osteogenesis, and angiogenesis and accelerate the process of osseointegration	Wang et al. (2021)
MAO and large-grit sandblasting	Ti	Micron-sized craters accompanied by sub-micron-scale pits, more porous architectures	BMMSC	dog	-	Enhance cell adhesion, proliferation, osteogenic and peri-implant new bone Formation and osseointegration	He et al. (2019)
MAO and SLA	Cp-Ti	A typical crater-like porous structure was formed on the surface	MC3T3-E1 cells			Enhance cellular attachment and proliferation	Kim et al. (2021)
MAO and HESP	Cp-Ti	Many non-uniform nanoparticles, some elliptical or circular micro-pores and some obvious cracks on MAO substrates	MC3T3-E1 cells	-	-	MC3T3-E1 cells on S-MAO substrate had better spreading, proliferation, and osteogenic differentiation capacities	Shen et al. (2020b)
MAO and HESP	Ti	Some porous structures and no obvious cracks	MC3T3-E1 cells	-	*S. aureus S. mutans*	Promoting the spreading, proliferation and differentiation of cells and having bactericidal effects on both bacterial	Shen et al. (2020a)
MAO and SFT	Ti3Zr2Sn3Mo25Nb alloy	Evenly distributed with sub-circular or elliptical crater-like and much fewer submicron pores	MC3T3-E1 cells			Nanocrystalline micro-arc oxidation coating is conducive to cell adhesion and growth and has good biocompatibility	Yu et al. (2022)
MAO and SLM	Ti-6AL-4V	The homogenous micro-/nano-porous oxide layer	Pre-osteoblast MC3T3-E1 cells	-	MRSA USA300	Highly porous SLM titanium bone implants that were biofunctionalized using PEO with Ag and Zn NPs in ratios of up to 75% Ag and 25% Zn fully eradicated bacterial inocula within 24 h in both *in vitro* and *ex vivo* experiments	Iaj et al. (2020)
MAO and SLM	Ti-10Ta-2Nb-2Zr alloy	Covered homogeneously with “volcano-like holes” that were micro/nano-diameter in scale. Large holes were encased in tiny holes and interconnected	MG-63 cells	rabbits	-	MAO coating and porous structure improved cell viability *in vitro* and increased new bone formation *in vivo*	Luo et al. (2021)
MAO and SG	Ti	Flower-like and spherical dense micro nanostructured deposits completely cover the surface	-	-	*E. coli*	The coating produced with MAO and sol-gel combined methods is doubly effective for both bioactive and antibacterial properties	Yılmaz (2021)
MAO and SG	Ti6Al4V alloy	A hierarchically rough structured topography with a large number of crater-like micropores	MC3T3-E1 cells	-	-	Good biocompatibility and enhancing cell proliferation	Li et al. (2020)
MAO and SG	Cp-Ti	The thickness and area of the coating increase, and the particle structure on the surface is covered, resulting in the disappearance of cracks	MC3T3-E1 cells	-	-	The composite coating has excellent anti-inflammatory and bone-promoting properties	Zhu et al. (2022)
MAO and UA	Cp-Ti	A relatively uniform brownish appearance, porous morphology and presence of depositions were observed	MC3T3-E1 cells	-	*S. aureus*	The introduction of ultrasonic vibration enhances the corrosion resistance, antibacterial capability and cellular response of the Cu-incorporated TiO2 coating	Zhang et al. (2021)
MAO and UA	Ti	Formation of an intact substrate/coating interface with the absence of large cracks	MC3T3-E1 cells	-	*S. aureus*	Both *in vitro* and *in vivo* biological examinations indicate the desirable biological response including biocompatibility and antibacterial property	Lv et al. (2021)
MAO and UA	Ti-Cu alloy	Increased the roughness	MC3T3-E1 cells	-	*S. aureus*	Improved the antibacterial activity and non-cytotoxicity but improved the early adhesion of MC3T3 cells	Hu et al. (2020)
MAO and bioactive factors	Ti6Al4V alloy	More pores on the metallic surface are covered by Ca, P coatings and HA-precipitated	murine bone marrow mesenchymal stem cell line D1 (mBMSCs)	rabbit	-	Ti-alloy implant modified by MAO with CaP-BMP2 is osteo-inductive and osteoconductive which can create better osteogenesis and angiogenesis	Teng et al. (2019)
MAO and bioactive factors	Cp-Ti	Petal sheets and roughness decreased	MC3T3-E1 cell		*E. coli*	The coating with BMP-2/chitosan/hydroxyapatite can significantly improve cell adhesion, spreading and proliferation with excellent antibacterial properties	Wang et al. (2019)

### 5.1 The combined application of MAO and hydrothermal method

By subjecting a material to high temperature and pressure, the hydrothermal method activates water molecules to dissolve or react with surface substances. This promotes crystal growth and alters the structure, morphology, and porosity of the material. Recent studies have shown that combining the hydrothermal method with MAO can result in the formation of micro/nano coatings on Ti and its alloys, which possess excellent biocompatibility and bone integration ability (Huang L. et al., 2021). Some studies also indicate that microwave-assisted hydrothermal technology combined with MAO, can regulate stem cell differentiation (Lin et al., 2020). Furthermore, applying HA coating on the Ti surfaces using MAO and hydrothermal steam treatment (SHT) not only shows good biocompatibility but also stimulates endothelial cells to secrete vascular endothelial growth factor, promoting the formation of capillary-like networks and inhibiting inflammation (Wang X. et al., 2021).

### 5.2 The combined application of MAO and SLA

SLA are commonly used method to improve the surface roughness of implants. However, using sharp alumina sand and caustic soda may not completely remove these materials, which may negatively affect bone formation around the implant and interfere with bone bonding. To solve this problem, some researchers have combined large particle sandblasting with MAO to create micro- and submicron-level 3D porous structures on Ti implants. This optimizes surface properties and improves new bone formation and bonding (He et al., 2019). Jinyoung Kim’s research on enhancing the surface roughness of Ti by combining sandblasting, acid etching and MAO. The typical crater-like porous structure can be observed under an SEM, which can help enhance the adhesion and differentiation of pre-osteoblasts, ultimately improving the overall biocompatibility (Kim et al., 2021).

### 5.3 The combined application of MAO and HESP

Different from sandblasting technology, shot peening (SP) technology surface cleaning can also enhance residual compressive stress and improve fatigue resistance. In a study conducted by Xinkun Shen et al., Ti substrates were treated with HESP and MAO to create porous TiO_2_ films containing Ca and P. The results showed that HESP pretreatment improved the stability of TiO_2_ coating and the bioactivity of MAO-treated samples (Shen et al., 2020c) Furthermore, the research revealed that using HESP-assisted MAO to produce Si/Cu-MAO coatings resulted in osteogenic properties and bactericidal activity against *S. aureus* and *S. mutans* (Shen et al., 2020b).

### 5.4 The combined application of MAO and SFT

SFT technology is widely used to refine the surface grain structure and improve the mechanical properties, as well as to improve the performance and life of biomaterials through severe surface plastic deformation. Yu Sen and other researchers combined SFT with MAO to form a uniform coating with nearly circular or oval crater-like pores of less than one micron on the surface of Ti3Zr2Sn3Mo25Nb alloy. This coating has demonstrated excellent biocompatibility and can promote the attachment and growth of cells (Yu et al., 2022).

### 5.5 The combined application of MAO and SLM

To achieve optimal new bone growth in porous scaffolds, it is crucial to maintain precise control and optimization of pore size, porosity, and connectivity throughout the fabrication process. Recently, SLM has garnered significant attention as an additive manufacturing technique for personalized orthopaedic implant production. Junsi Luo’s research team leveraged SLM technology to precisely manufacture Ti alloy implants, using MAO technology to modify their surface for micro/nano-level roughness before coating them with HA. These novel HA coatings possess appropriate mechanical properties, excellent biocompatibility and bioactivity, and enhanced cell viability *in vitro*, as well as new bone formation *in vivo*. They may be a promising alternative treatment option for high-risk or younger patients in need of more durable implants (Luo et al., 2021). In another study, researchers synthesized porous Ti implants using SLM and functionalized them with Zn and Ag nanoparticles embedded through MAO with Ti oxide layers, effectively preventing implant-associated infections (IAI) caused by antibiotic-resistant bacteria (Iaj et al., 2020).

### 5.6 The combined application of MAO and Sol-gel coating method

Researchers have utilized a sol-gel coating method capable of forming uniform nanofilms on the material surface, resulting in bioactive antimicrobial polymer/Ca-P bio-composites on MAO-modified titanium for dental and orthopaedic implant coatings (Yılmaz, 2021). The findings suggest that the coating promotes cell recruitment and improves cell proliferation during the initial stages of culture, potentially extending the lifespan of the implant (Li R. et al., 2020). To improve the performance of oral implants, researchers used MAO and sol-gel techniques to fabricate a multifunctional composite coating consisting of silica particles and zirconium hydrogen phosphate on the Ti surface, which exhibits higher friction and corrosion resistance, as well as better anti-inflammatory and osteogenic properties (Zhu et al., 2022).

### 5.7 The combined application of MAO and UV

UV is a commonly used auxiliary technique to tune the morphological and structural properties of MAO coatings to enhance their performance. Ultrasound-assisted micro-arc oxidation (UMAO) is a promising surface modification method for fabricating high-quality titanium-based orthopaedic implants. Studies have shown that the use of UV in the MAO process can increase the corrosion resistance and bonding strength of TiO_2_ coatings, improving their biological properties (Lv et al., 2021a). UMAO can also be used to enhance the corrosion resistance, antimicrobial properties, and cytocompatibility of Cu-doped TiO_2_ coatings, creating functional surfaces with higher uniformity and performance for biomedical materials (Zhang et al., 2021d). Additionally, UMAO can modify the surface of Ti-Cu alloys to improve their antibacterial properties and cell biocompatibility (Hu et al., 2020).

### 5.8 The combined application of MAO and bioactive factors

Recent research has shown that combining MAO with other bioactive elements such as Bone Morphogenetic Proteins (BMPs), can stimulate new bone formation. However, the effectiveness of BMPs in promoting osteogenesis depends on the careful control of carrier type and release rate. In a study conducted by Teng et al., a unique approach was employed involving 3D printing, MAO treatment, and the coprecipitation of calcium and P layers with BMP-2 technology to fabricate porous titanium alloy-based implants featuring interconnected channel structures.

The growth factor BMP-2 exhibited a continuous diffusion pattern from the central region to the periphery of the implant, thereby facilitating the proliferation, differentiation, and mineralization of bone cells. *In vivo* experiments further revealed the infiltration of bone tissue and blood vessels into the central area of the implant. It was observed that MAO-CaP-BMP-2 outperformed both the MAO and MAO-CaP groups in terms of promoting new bone formation, underscoring the potential of MAO-CaP-BMP2 in enhancing bone healing. As a result, MAO-CaP-BMP2 stands as a promising candidate for applications as a growth factor vector. (Teng et al., 2019).

In addition, some studies have found that a petal-like HA/TiO_2_ composite coating was prepared on the Ti surface through one-step MAO, and then pure chitosan (CS) was used and BMP-2 respectively loaded with CS coatings on the HA/TiO2 surface using a dip coating method, which can give Ti good antibacterial and biological properties, and the thicker the HA layer, the higher the load of BMP-2 and CS. The larger the amount, the better the bonding strength, antibacterial activity and biocompatibility between coatings. (Wang et al., 2019b).

To sum up, the combined MAO technology with other surface treatment methods has demonstrated immense potential in improving surface properties and enhancing the biological performance of biomedical materials. The integration of MAO with diverse techniques such as hydrothermal method, SLA, HESP, SFT, SLM, sol-gel coating method, UV and bioactive factors has resulted in the development of novel coatings that possess superior biocompatibility, antibacterial properties, corrosion resistance, and osteogenic properties, thereby making them well-suited for orthopaedic and dental implant applications. These advancements hold great promise for enhancing the long-term outcomes of implant surgeries while mitigating associated risks. Continued research and development of these hybrid technologies could lead to further improvements in the field of biomedical surface modification, ultimately benefiting patients worldwide.

## 6 Limitations and future scope

MAO coatings have shown tremendous potential for medical applications, particularly in titanium and its alloys. These coatings enhance surface properties such as biocompatibility, corrosion resistance, wear resistance, and hardness, making them attractive for improving the performance and safety of medical devices. Unfortunately, the use of MAO coatings in medical applications is not without limitations. One major issue is that the coating process can be both expensive and complex, thereby limiting its widespread use. Additionally, the uniformity and thickness of the coating may vary depending on the materials used and the process parameters applied, which can affect the overall performance and reliability of the coating material. Furthermore, while the porous morphologies of MAO coatings theoretically can enhance the bonding between implants and bone tissue, promoting bone tissue growth, the porosity may create channels for accelerated corrosion ion (Cl^−^) penetration, thus limiting the application of some biodegradable materials. Therefore, researchers exploring the use of magnesium (Mg) in the biomedical field tend to produce MAO coatings with self-sealing pores.

The parameters of MAO are crucial for producing coatings with excellent performance. Process parameters include electrolyte composition, voltage, current density, frequency, response time, and duty cycle. Parameter control aims to obtain a surface that is uniform, stable, adhesive, and bioactive. Although preliminary research has shown promising results, our understanding of the long-term biocompatibility of MAO coatings remains limited. Further research is needed to evaluate their durability and biocompatibility over time. Regarding antibacterial applications, MAO coatings have great potential to reduce bacterial adhesion and growth. However, the efficacy of these coatings depends on several factors, such as coating thickness, roughness, and bacterial strain. Incorporating high concentrations of metal ions into the coating may have adverse effects on cell biocompatibility, and suitable concentrations need to be investigated. Additionally, further research is needed to explore the mechanisms by which these coatings affect cells and bacteria (Zhang et al., 2018b). Furthermore, MAO may need to be combined with other antibacterial agents and treatments to achieve optimal results. Photodynamic antibacterial therapy is receiving increasing attention due to its bactericidal efficiency and safety being higher than those of metal ions. For example, Han et al. found that a MoS2-modified TiO_2_ coating prepared using a hybrid process exhibits excellent antibacterial activity when exposed to 808 nm near-infrared light (Han et al., 2021). Scholars also indicate that functional TiO_2_ coatings co-doped with nitrogen and bismuth achieve antibacterial effects under visible light with good biocompatibility and reactivation potential (Nagay et al., 2019).

Additionally, Wenyue Yang et al. reported that TiO_2_ nanoparticles can be incorporated into the β titanium alloy Ti-35Nb-2Ta-3Zr (TNTZ) matrix using friction stir processing (FSP) as a bioactive reinforcement, which can build an integrated micro-nano composite layer that facilitates the adhesion and proliferation of BMSCs (Yang et al., 2023). Therefore, MAO technology can be considered in the future to build micro-nanostructures on the surface of titanium alloys.

In summary, despite these limitations, researchers continue to explore methods for optimizing coatings, developing new materials and formulations, and combining MAO with other physical and chemical methods to improve its biocompatibility, antibacterial properties, and other properties. This technology has tremendous potential in enhancing the safety and performance of medical biomaterials made from titanium and its alloys.

## 7 Conclusion

In this review, we delve into recent advancements in the biomedical application of MAO coatings for Ti and its alloys. We focus specifically on process parameters, biocompatibility, antibacterial properties and combinations with other technical aspects. Our conclusions are summarized below:

1) We analyse various process parameters such as electrolyte composition, voltage, and current, that can influence the characteristics of MAO coatings, including their surface roughness, porosity, structure, and morphology attributes.
2) We emphasize the importance of studying the biocompatibility of MAO coatings for Ti and its alloys. Our findings suggest that these coatings have significant potential to enhance cell compatibility, blood compatibility, corrosion resistance, angiogenesis activity, osteogenic activity, and bone integration.
3) Another aspect we highlight is the incorporation of metal elements, such as Cu, Ag, and Zn into MAO coatings. This integration is proven to be an effective technique for imparting antibacterial properties to Ti and its alloys.
4) We discuss several pretreatment and posttreatment hybrid technologies developed to improve the biological properties of MAO coatings. The combination of MAO with hydrothermal methods, SLA, HESP, SFT, SLM, the sol-gel coating method, UV, and bioactive factors provide advantages over using individual techniques.
5) Finally, while considerable progress has been made in researching bioactive MAO coatings on Ti and its alloys, several challenges still need to be addressed. Specifically, further understanding of the mechanism of MAO is needed to facilitate its wider adoption in the industry.

In our review, we emphasize the enormous potential of micro-arc oxidation coatings in improving performance and expanding the applications of titanium and its alloys. In addition, it emphasizes the continuous necessity of MAO technology research in the field of biomedicine. To further promote the application of Ti and its alloys in medical applications, we suggest that future researchers focus on real-world application scenarios, delve into the complex mechanisms behind MAO coatings, and strive to advance this field.

Reducing the gap between laboratory findings and actual medicine is the future research direction. Understanding the complex interactions between MAO coatings and biological systems at the molecular level not only helps to develop customized coatings but also promotes the safe and effective integration of titanium-based materials in the human body. In addition, collaborative efforts bringing together materials scientists, biologists, medical practitioners, and engineers will play a crucial role in promoting the transformation of MAO-coated titanium and its alloys into innovative medical devices and implants. These interdisciplinary methods can bring breakthroughs, redefine the prospects of medical technology, provide enhanced treatment for patients, and improve their quality of life.

In summary, our review serves as a catalyst, and it highlights the potential and necessity for research in the field of micro-arc oxidation coatings on titanium and its alloys. It is through joint efforts and a deeper understanding of basic science that we can fully utilize the capabilities of MAO technology, completely transform biomedical applications, and usher in a new era of medical innovation.
