# Supplementary material for: Micro-arc oxidation (MAO) and its potential for improving the performance of titanium implants in biomedical applications
Source: Front Bioeng Biotechnol. 2023 Nov 7;11:1282590. doi: 10.3389/fbioe.2023.1282590 (PMC10662315; doi:10.3389/fbioe.2023.1282590)
Supplement: Supplementary file 1 [file Table1.docx]

**Table S1**

Antibacterial tests and results on MAO-modified Ti-surfaces bearing single or multiple elements.

| Agent | Substrate | Electrolyte | Bacterial Species | Duration (h) | Test Inoculum (CFU/ml) | Planktonic/ Adherent | Antibacterial rates | Antibacterial mechanism | Biocompatibility | Main Outcomes | Ref. |
| --- | --- | --- | --- | --- | --- | --- | --- | --- | --- | --- | --- |
| Cu | Ti | 0.02 M Copper gluconate | S. aureus | 24 | 10^4^ | Adherent | 100% | Contact | YES | After 24 h: 100% antibacterial rate on Cu surfaces Morphological changes and disrupted membrane of bacterial cells | (Zhao et al., 2019) |
| Cu | Ti | 10 g/L Na2Cu-EDTA | S. aureus | 24 | 10^5^ | Adherent | - | Release | YES | After 24 h: more dead bacteria on Cu surface. Shape changes and membrane disruption of bacteria cells | (Zhang et al., 2020d) |
| Cu | CP-Ti | 300g/450g/600g Cu(NO3)2·3H2O | C.albicans  E. coli | 24 | 10^8^ | Planktonic  Adherent | - | Contact | - | After 24h: Less bacteria adhered on the Cu surface. | (Rokosz et al., 2020) |
| Cu | Ti-6Al-4V alloys | 10 g L^-1^ EDTA-CuNa2(Cu-1.01wt%/Cu-1.92wt%) | S. aureus  E. Coli | 24 | 5x10^5^ | Planktonic  Adherent | 100% | Contact | YES | All coatings demonstrate short and long-term antibacterial ability against S. aureus and E. coli. | (Liang et al., 2020) |
| Cu | CP-Ti | 0.2 mol•L^-1^ Cu（CH3COO）2 | S. aureus | 24 | 10^5^ | Adherent | ＞99% | Contact | YES | After 24 h: Bacteria on the surface of MAO coatings containing Cu are reduced by >99%. | (He et al., 2020) |
| Cu | CP-Ti | 0.2 or 2 mM CuSO4·5H2O | S. aureus | 2 | 1x10^7^ | Adherent | - | Contact | YES | After 2h: the macrophages cultured on Cu-MAO surface phagocytized more bacteria and exhibited a higher bacterial killing rate | (Huang et al., 2018) |
| Cu | CP-Ti | 0.3 Cu and 3.0 Cu (at%) nanoparticles | S. aureus | 24 | 1x10^7^ | Adherent Planktonic | - | Contact | YES | After 24: significant reduction in bacterial colonies on Cu NPs coatings on 0.3 Cu and 3.0 Cu killing all adhered bacteria; bacterial lysis and cytoplasmic leakage have been observed. | (Zhang et al., 2018b) |
| Cu | Ti | 0.025/0.05/0.075mol/L Cu(CH3COO)2· 2H2O | S. aureus  S. mutans | 24 | 1x10^6^ | Adherent | S. aureus: 99.1 ± 2.3%  S. mutans: 98.7 ± 3.5% | Contact | YES | After 24h: Si/Cu-MAO exhibited significant bacteriostatic efficacy against S. aureus and S. mutans, the majority of bacteria on Si/Cu-MAO surfaces were stained red (dead bacteria) | (Shen et al., 2020) |
| Cu | CP-Ti | 2 g/5 g/10 g of Na2Cu-EDTA | E. coli | 24 | 10^5^ | Planktonic | 2 g Na2Cu-EDTA: 68%  5 g Na2Cu-EDTA: 60%  10 g Na2Cu-EDTA: 68% | Contact | YES | After 24h: all coating cooperated with Cu have the ability of antibacterial, there was lysed appearance of bacteria with ruptured cell membranes. | (Zhang et al., 2021a) |
| Cu | CP-Ti | 0.5, 1.0 and 2.5 mM CuCl2 | S. aureus  E. coli | 24h | 3-5×10^6^ | Planktonic | - | Contact | YES | After 24h: The numbers of both E. coli and S. aureus on the samples containing Cu were smaller than those on the control samples without Cu | (Shimabukuro et al., 2020b) |
| Ag | CP-Ti | 0.025 mol/L Ag2O NPs, AgO NPs | S. aureus | 24h | 10^4^ | Planktonic | ＞99% | Contact  Release | YES | After 24h: both AR of the coatings with Ag2O NPs or AgO NPs were over 99%. After 4w: the AR decreased to 83.9% and 35.4% respectively | (Zhang et al., 2021b) |
| Ag | CP-Ti | 1 g/L AgNO3 | S. aureus | 24h | NR | Planktonic | 45% | Contact | YES | After 24h: The number of S. aureus reduced significantly in the extracts of the Ag-CP coatings. | (Sedelnikova et al., 2019) |
| Ag | Ti | 0.03 M AgNO3 | S. aureus | 24 | 10^5^ | Planktonic | - | - | YES | After 24: The live bacteria adhering to the surfaces of the porous Si/Ag-TiO2 coating had almost disappeared, and they were all red (dead) bacteria. | (Zhao et al., 2022) |
| Ag | Ti | 0.01/0.02/0.05 mol/L Ag2O NP | S. aureus | 24 | 10^5^ | Planktonic | 0.01mol/L Ag2O NP: 75%  0.02mol/L Ag2O NP: 90%  0.05mol/L Ag2O NP: 99% | Contact | NO | After 24h: Ag-incorporated TiO2 coatings show the strong anti-bacterial ability. And Ag contents of TiO2 coatings lead to lower numbers of bacterial colonies. | (Lv et al., 2019) |
| Ag | CP-Ti | 0.1 g/L, 0.5 g/L and 1.0 g/L AgNPs | E. coli  S. aureus | 24 | NR | Adherent | - | Contact | - | After 24h: Samples with high silver content (0.5 g/L and 1.0 g/L AgNPs) exhibited a complete reduction of E. coli and a 6-log reduction of S. aureus. | (Thukkaram et al., 2020) |
| Ag | Ti | 0.04 g/L, 0.08 g/L, 0.17 g/L, and 0.34 g/L of AgNO3 | S. aureus | 24h | 1 × 10^5^ | Planktonic  Adherent | 0.17, 0.34 g/L of AgNO3: both 100% | Contact  Release | YES | After 24h: Bacterial inhibition zones appeared on the surface of all MAO coatings containing Ag. The AR of Sr/Ag0.17 (0.58 wt% Ag) and Sr/Ag0.34 (1.29 wt% Ag) both were 100%. After 30-days PBS immersion, considerable antibacterial activity is retained and AR still in high level. | (Zhang et al., 2021c) |
| Ag | CP-Ti | 180 mg/L Ag nanoparticles | S. aureus | 2,4,6,24 | 10^6^ | Planktonic  Adherent | - | Contact  Release | YES | After 2,4,6h: AgNP-PEO Ti could inhibit bacterial adhesion and surface biofilm formation by S. aureus. And bacterial cell amounts after cocultivation with AgNP-PEO Ti were lowest | (Oleshko et al., 2020b) |
| Ag | CP-Ti | 0·0005, 0·001 and 0·002  mol  L−1 AgC2H3O2 | S. aureus | 24 | 2·5 × 10^5^–10^6^ | Planktonic | 0·0005,0·001 and 0·002 mol L^-1^ AgC2H3O2:36.66,99.98,99.98% | Contact | YES | After 24h: Ag incorporated MAO coating exhibited superior antibacterial characteristics against S. aureus. | (Teker Aydogan et al., 2018) |
| Ag | CP-Ti | 0-10 mM AgNO3 | E. coli  S. aureus | 24 | (0.4−3.0) × 10^8^ | Planktonic | - | Contact | YES(≤0.5mM)  NO(＞0.5mM) | After 24h: the bacterial colonies formed were less on the surface of the MAO-treated Ti samples in the electrolyte with 0.05 mM or higher Ag concentrations. | (Shimabukuro et al., 2019) |
| Ag | Ti | One step method: 0.00025 mol/L AgNO3  Two step method: 0.0003 mol/L AgNO3 | E. coli | 24 | 10^6^ | Planktonic | ＞99% | Contact | YES | After 24 h: the growth of E. coli on both Ca/P/Ag coatings was inhibited, and after culture with E. coli for 24 h, almost all the bacteria died. | (Zhang et al., 2020b) |
| Zn | Ti-15Mo alloy | 10 g∙L−1 ZnO (Zn1)  25 g∙L−1 Zn3(PO4)2（Zn2）  10 g∙L−1 Zn3(PO4)2 (Zn3) | S. aureus  clinical S. aureus  reference S. epidermidis  clinical S. epidermidis | 4 | 10^6^ | Adherent | - | Contact | YES | After 24h: The MAO coating surface with Zn exhibited higher adhesion of S. aureus and MRSA, while displaying a lower count of S. epidermidis. | (Hu et al., 2022) |
| Zn | TiZrNb alloy | ZnO nanoparticles (40 g/L) | S. aureus | 2,4,6,24 | 10^5^ | Planktonic  Adherent | - | Contact | YES | After 6h: PEO treated coating with ZnO NPs had higher antibacterial activity. | (Oleshko et al., 2020a) |
| Zr | Ti | 1/3/5 g/L ZrO2 NPs | S. aureus | 24h | 1 × 10^5^ | Planktonic  Adherent | 1,3,5g/L ZrO2: 81.55%, 86.80%, 94.37% | Contact | YES | The incorporation of ZrO2 NPs in the coatings had a positive effect on their antibacterial properties. An increase in ZrO2 NPs concentration from 1 to 5 g/L led to an increase in the antibacterial rate of coatings from 81.55 to 94.37% against S. aureus bacteria within 24 h. | (Molaei et al., 2022) |
| Zr | Ti–6Al–4V | 1/3/5 g/L ZrO2 NPs | S. aureus  S. epidermidis  E. coli  P. aeruginosa | 3 | 10^5^ | Planktonic | - | NR | - | After 3h: MAO coating without ZrO2 showed the best antibacterial effect, reducing the growth of E. coli and S. epidermidis by 100%, and achieving a growth inhibition rate of 51% and 7% for S. aureus and P. aeruginosa bacteria, respectively. As the concentration of ZrO2 increases, the antibacterial effect of the coatings decreases, possibly due to changes in surface roughness, pore size, and surface wettability. | (Nikoomanzari et al., 2020) |
| B | CP-Ti | 0.02 M Na2B4O7·10H2O | S. aureus  P. aeruginosa | 6, 24h | NR | Planktonic | - | Contact | YES | After 24h: A significant reduction in the number of live bacteria, and an increase of dead bacteria on the coating containing boron | (Sopchenski et al., 2018) |
| Mn | CP-Ti | 0.01 mol/L Na2MnEDTA | S. aureus | 24 | 10^5^ | Planktonic | 70.8% | Contact  Release | YES | After 24h: More dead bacteria, damaged membranes of cells, and less adhered bacteria can be clearly observed on the surface of the Mn incorporated MAO coating. | (Zhang et al., 2020c) |
| W | Ti6Al4V | 4 g/L Na2WO4.2H2O | E. coli  S. aureus | 24 | 10^5^ | Planktonic  Adherent | ＞99% | Contact | - | After 24h: The W-containing MAO coating significantly inhibited the adhesion of bacteria and reduced the quantities of planktonic bacteria in culture medium. | (Zhou et al., 2019) |
| Y(Yttrium) | Ti6Al4V | .03 M, 0.06 M and 0.09 M of yttrium acetate (C6H9O6Y.xH2O) | S. aureus  E. coli | 12 | - | Planktonic | - | Contact | YES | After 12h: MAO coatings prepared with electrolyte incorporating 0.09 mM Y acetate showed a strong antibacterial effect, as evidenced by the presence of cell debris from both S. aureus and E. coli on the coated surface and a significant decrease in the number of surviving bacteria accompanied by an increase in the number of dead bacteria. | (Zhang et al., 2020a) |
| Ga(gallium) | CP-Ti | 1g Ga2O3 microparticles | S. aureus  E. Coli | 24h | 1×10^6^ | Planktonic | S. aureus＞99.6±2.2%  E. Coli＞96.5±1.7% | Contact | YES | After 24h: Few colonies are observed on MAO coatings with Ga, which have excellent antibacterial effect. | (F et al., 2022) |
| Ag  Cu | CP-Ti | 2.5 mM silver nitrate  2.5 mM copper chloride | E. coli | 24h | 1x10^6^ | Planktonic | - | Contact | - | After 24h: both Ag-and Cu-incorporated specimens developed antibacterial effects against E. coli.  After 28d: Silver-doped samples' antibacterial effect weakened significantly, while copper-doped samples remained unchanged. | (Shimabukuro et al., 2020a) |
| Ag  Zn | CP-Ti | 5 g/L Na2Zn-EDTA  5 g/L Ag nanoparticles | S. aureus | 24h | 10^5^ | Adherent | Zn:92%  Ag:96%  Ag+Zn:98% | Contact | YES | After 24h: All MAO coatings were covered by large amounts of dead bacteria. And S. aureus colonies reduced. Antibacterial capacities of those TiO2 coatings follow the decreasing order: Ti-Ag-Zn-EDTA > Ti-Ag > Ti-Zn-EDTA. | (Lv et al., 2020) |
| Ag  Zn | CP-Ti | 0.01M ZnO  0.01M Ag | S. aureus | 24h | NR | Planktonic | ZnO:87%  Ag NPs:96%  Ag NPs+ZnO:98% | Contact | YES | After 24h: the surface of titanium coating modified by ZnO and Ag NPs appeared high intensities of red fluorescence (dead bacterial colonies). And the AR of the coating of co-incorporation of both Ag and Zn is ~98%. | (Lv et al., 2021) |
| Cu  Zn | Ti–6Al–4V alloy | EDTACuNa2(g/ L):2/6/10  EDTAZnNa2 (g/L):2/6/10 | MRSA  E. coli  S. aureus | 24 | 0.2x10^6^ | Planktonic | - | Contact | YES | After 24h: the MAO-treated samples modified by Cu and Zn possess superior antibacterial properties and nearly no bacterial appeared in the surface compared to the control. | (Wang et al., 2020) |
| Cu  Zn | CP-Ti | 0.0013 M copper acetate  0.005,0.01,0.02,0.04 M zinc acetate | S. aureus | 6, 24 | 10^5^ | Planktonic | - | Contact  Release | YES | After 6 and 24h: the attached number on the Cu and Zn doped surfaces less than the control group samples  After 24h: Viable bacteria adhered on the Cu and Zn doped surfaces are reduced, and the dead ones stained in red can be obviously observed. | (Zhang et al., 2018a) |
| Zr  Zn | Ti–6Al–4V alloy | ZrO2 (5 g/l)  ZnO (5 g/l) | S. aureus | 3, 6 | 10^6^ | Planktonic | 3h/6h: PEO + ZnO:50%/85%  PEO + ZrO2:45%/70%  PEO + ZrO2 + Zn：55%/90% | Contact | - | After 6h: The employment of mix ZrO2–ZnO nanoparticles to the PEO coating was very effective in inhibiting S. aureus activity (90%). | (Nadimi and Dehghanian, 2021) |
